# Predicting antidepressant response by monitoring early improvement of individual symptoms of depression: individual patient data meta-analysis

**DOI:** 10.1192/bjp.2018.122

**Published:** 2018-06-28

**Authors:** Ymkje Anna de Vries, Annelieke M. Roest, Elisabeth H. Bos, Johannes G. M. Burgerhof, Hanna M. van Loo, Peter de Jonge

**Affiliations:** 1 Interdisciplinary Center Psychopathology and Emotion Regulation, Department of Psychiatry, University Medical Center Groningen, University of Groningen, the Netherlands; Division of Developmental Psychology, Department of Psychology, University of Groningen, the Netherlands; 2 Department of Epidemiology, University Medical Center Groningen, University of Groningen, the Netherlands; 3 Interdisciplinary Center Psychopathology and Emotion Regulation, Department of Psychiatry, University Medical Center Groningen, University of Groningen, the Netherlands; 4 Interdisciplinary Center Psychopathology and Emotion Regulation, Department of Psychiatry, University Medical Center Groningen, University of Groningen, the Netherlands; Division of Developmental Psychology, Department of Psychology, University of Groningen, the Netherlands; 5 Interdisciplinary Center Psychopathology and Emotion Regulation, Department of Psychiatry, University Medical Center Groningen, University of Groningen, the Netherlands; Division of Developmental Psychology, Department of Psychology, University of Groningen, the Netherlands; 6 Interdisciplinary Center Psychopathology and Emotion Regulation, Department of Psychiatry, University Medical Center Groningen, University of Groningen, the Netherlands; Division of Developmental Psychology, Department of Psychology, University of Groningen, the Netherlands

**Keywords:** Antidepressants, depression, early improvement, treatment response, individual patient data meta-analysis

## Abstract

**Background:**

Improvement in depression within the first 2 weeks of antidepressant treatment predicts good outcomes, but non-improvers can still respond or remit, whereas improvers often do not.

**Aims:**

We aimed to investigate whether early improvement of individual depressive symptoms better predicts response or remission.

**Method:**

We obtained individual patient data of 30 trials comprising 2184 placebo-treated and 6058 antidepressant-treated participants. Primary outcome was week 6 response; secondary outcomes were week 6 remission and week 12 response and remission. We compared models that only included improvement in total score by week 2 (total improvement model) with models that also included improvement in individual symptoms.

**Results:**

For week 6 response, the area under the receiver operating characteristic curve and negative and positive predictive values of the total improvement model were 0.73, 0.67 and 0.74 compared with 0.77, 0.70 and 0.71 for the item improvement model. Model performance decreased for week 12 outcomes. Of predicted non-responders, 29% actually did respond by week 6 and 43% by week 12, which was decreased from the baseline (overall) probabilities of 51% by week 6 and 69% by week 12. In *post hoc* analyses with continuous rather than dichotomous early improvement, including individual items did not enhance model performance.

**Conclusions:**

Examining individual symptoms adds little to the predictive ability of early improvement. Additionally, early non-improvement does not rule out response or remission, particularly after 12 rather than 6 weeks. Therefore, our findings suggest that routinely adapting pharmacological treatment because of limited early improvement would often be premature.

Antidepressants are first-line treatments for major depressive disorder (MDD).^1^^–^^3^ However, many patients fail to respond, with response rates averaging around 50% in clinical trials,^4^ and it is important to identify these patients as soon as possible to minimise the duration of ineffective treatment and the time until response. Clinical guidelines currently recommend 4–8 weeks of treatment before considering a change in treatment in patients who show no improvement,^1^^–^^3^ although the evidence base for this recommendation is limited. Antidepressant effects can be detected within the first treatment week,^5^ and numerous studies show that early improvement is associated with later response or remission.^6^^–^^10^ However, these studies disagree on whether lack of early improvement justifies a change in management. For instance, one study found that only 4% of participants with minimal improvement after 2 weeks reached remission by week 4,^7^ but in another study, 44% of participants without early improvement still responded after 12 weeks of treatment.^8^ On average, most studies indicate that at least 20–30% of participants without early improvement attain response or remission after 4–12 weeks of treatment, which is reduced from the overall probability of around 50%, but is not negligible.^9^^,^^10^ Conversely, many early improvers do not achieve such outcomes. Hence, better predictive models are desirable.

One possibility to extend these models is to examine individual symptoms, rather than only the total depression score. There are meaningful differences between symptoms (e.g. regarding risk factors and disability^11^), and severity of specific symptoms is associated with prognosis.^12^^,^^13^ Previous studies have found that response can be predicted by early improvement in several symptoms, including depressed mood, somatic symptoms and loss of insight.^14^^–^^19^ However, these studies did not investigate whether improvement in individual symptoms is more informative than improvement in the total score alone – something we therefore investigated in this study. We also examined whether there are interactions between early-improving symptoms, gender and age. Finally, we examined whether individual symptoms are differentially predictive for response to different antidepressant classes.

## Method

### Data source and trial selection

We requested individual patient data from Clinical Study Data Request,^20^ a data-sharing platform providing data from (among others) trials of antidepressants developed by participating sponsors (GlaxoSmithKline and Lilly). We examined second-generation antidepressants (SGAs, defined as selective serotonin reuptake inhibitors (SSRIs), serotonin–norepinephrine reuptake inhibitors (SNRIs) or other antidepressants approved after 1987) because older antidepressants are considered second-line options. However, we also included trials of new chemical entities that have never been approved for treatment of MDD, if an approved SGA was used as an active comparator. We included randomised, placebo- or active-comparator-controlled double-blind trials for MDD in adults, which had a minimum duration of 6 weeks and used the Hamilton Rating Scale for Depression (HRSD) at baseline, week 2 and either week 6 (±1) or week 12 (±1) (or both). We excluded trials that specifically included only participants with additional symptoms (e.g. MDD with pain).

### Patient population

We only included participants assigned to placebo or SGAs. No eligible trials included participants assigned to non-SSRI/SNRI SGAs (e.g. mirtazapine), so our final sample consisted of participants assigned to placebo, SSRIs or SNRIs. We took a complete-case approach, only including participants who had valid HRSD scores at baseline, week 2 and week 6 or 12. Week 2 visits took place on day 14 (±7 days), week 6 visits on day 42 (±14 days) and week 12 visits on day 84 (±14). If a participant had multiple visits within the eligible time frame, we selected the visit closest to the intended visit day or, if eligible visits were equally close (e.g. day 35 and day 49), we randomly selected one of the visits.

### Training and test data

We randomly split the data into an 80% training set and 20% test set, stratified by treatment group (placebo, SSRI or SNRI). The training set was used for model discovery and cross-validation, and prediction accuracy was assessed in the test set.

### Outcomes and predictors

Our primary outcome was response (≥50% reduction in HRSD (17-item version) score) at week 6.^21^ Secondary outcomes were remission (score of ≤7 on the HRSD 17-item version) at week 6, and response and remission at week 12. We chose response as our primary outcome rather than remission because we believed it to be a more realistically achievable outcome by week 6, because it is less dependent on baseline severity and because it is commonly used as the primary outcome in other meta-analyses.^4^^,^^22^

Improvement in symptoms was calculated from the baseline and week 2 HRSD items and dichotomised into no improvement (no change or worsening) or improvement (improvement in item score of 1 or more) for each individual symptom. Baseline HRSD items were dichotomised into absent (score of 0) or present (score of ≥1). Early improvement in the total HRSD score was dichotomised into no improvement (<20% improvement) or improvement (≥20% improvement), consistent with other studies,^9^ whereas the baseline HRSD score was standardised. As demographic variables, we included age (standardised) and gender.

### Statistical analysis

Our primary analyses only included antidepressant-treated participants. For variable selection, we used least absolute shrinkage and selection operator (lasso) logistic regression,^23^ implemented in the glmnet package (version 2.0–5) for R software (version 3.3.0). For each outcome (response and remission at week 6 and 12), we built four models: a baseline model, including only baseline HRSD score, HRSD items, age and gender; a total improvement model, including these baseline variables and early improvement in the total HRSD score; an item improvement model, including all these variables and early improvement in the 17 HRSD items; and an item interactions model, including all of the above and all two-way interactions among early-improving items, age and gender. The optimal regularisation penalty (*λ*) was determined by ten-fold cross-validation. We favoured sparser models by choosing the largest *λ* whose deviance was within one s.e. of the minimal deviance.^24^

From each lasso model, we selected all variables with non-zero coefficients to build a mixed-effects logistic regression model with a random intercept for trial, using the lme4 package (version 1.1–12). Hence, we built four separate mixed-effects models for each outcome.

### Model performance

The prediction accuracy of each mixed-effects model was assessed in the test set by determining the area under the receiver operating characteristic curve (AUC) (R package pROC, version 1.8). The model with the highest AUC was considered the best model. We also determined the accuracy, sensitivity, specificity, positive predictive value (PPV) and negative predictive value (NPV) for each model by assigning participants with a model-predicted probability of response/remission of ≥50% to the response/remission group.

### Secondary analyses and *post hoc* analyses

We performed secondary analyses in the total group of antidepressant- and placebo-treated participants. In these analyses, we included treatment group (placebo versus SSRI versus SNRI) as a predictor to examine whether associations between early-improving items and outcome were dependent on antidepressant class (suggesting a drug-specific mechanism).

In our main and secondary analyses, we dichotomised early improvement, for comparability with other studies. However, we conducted additional *post hoc* analyses in which baseline item scores and early improvement (change from baseline in the total score and the individual items) were included as continuous variables.

To examine the influence of taking a complete-case approach, we performed single imputation of participants with a valid baseline and week 2 visit, using the *mice* package for R. We used predictive mean matching to predict HRSD score at week 6 or 12, using as predictors all variables also included in our main analyses. We repeated our main analyses in the imputed data-set.

### Ethics statement

Ethical approval was not required for this study as it used only de-identified patient data. The study is registered with Clinicaltrials.gov, under the identifier NCT02934035.

## Results

### Trials and patients

We requested and received data for 32 trials. However, two trials proved to be ineligible (no week 6 or 12 visit). The remaining 30 trials investigated duloxetine (15 trials), paroxetine (13 trials) or new chemical entities (two trials). Thirteen trials also included other SGAs (escitalopram, fluoxetine, paroxetine or venlafaxine). These 30 trials included 10 365 participants, of whom 8242 had a week 6 visit. The ten trials with a duration of ≥12 weeks included 4487 participants, of whom 3103 had a week 12 visit. Sample characteristics are shown in Table 1. Supplementary Table 1, available at https://doi.org/10.1192/bjp.2018.122, provides further details about the individual trials.

**Table 1:** Sample characteristics

	Week 6 sample			Week 12 sample		
	Placebo	SSRI	SNRI	Placebo	SSRI	SNRI
Sample size	2184	3322	2736	652	1270	1181
Mean baseline HRSD score (s.d.)	21.5 (4.7)	22.1 (4.2)	20.8 (4.7)	22.2 (4.5)	22.8 (3.5)	22.1 (4.6)
Mean age (s.d.)	44 (14)	43 (14)	45 (14)	50 (16)	45 (14)	48 (15)
Gender, % female	63.5	61.8	65.7	64.7	62.7	65.6
Early improvers, %	52.7	62.9	62.3	54.4	63.1	66.3
Responders, %	38.3	52.4	49.9	53.2	69.4	67.2
Remitters, %	22.6	32.1	32.7	34.5	49.4	48.9

HRSD, Hamilton Rating Scale for Depression; SNRI, serotonin–noradrenaline reuptake inhibitor; SSRI, selective serotonin reuptake inhibitor.

### Variable selection

Variable selection was performed in the training data-set. Detailed information about the variables selected by the lasso regressions are provided in Supplementary Tables 2–5. In brief, all improvement models selected early improvement in the total score. The item improvement models generally selected most of the early-improving HRSD items. However, items 3 (suicide) and 15 (hypochondria) were never selected, whereas items 1 (depressed mood), 2 (guilt), 4 (early insomnia), 7 (work and activities), 10 (psychological anxiety) and 13 (general somatic symptoms) were always selected. Baseline HRSD items were selected infrequently. All item interaction models selected a number of interactions among early-improving symptoms; additional interactions between early-improving symptoms and age or gender were only selected by the item interaction model for remission at week 12.

### Model performance

Model performance was assessed in the test data-set. For response at week 6, the baseline model performed quite poorly (AUC 0.60). The total improvement model performed significantly better (AUC 0.73), and the item improvement and item interactions model performed similarly (AUC 0.77) and significantly better than the total improvement model. For remission at week 6 and response and remission at week 12, the patterns were similar, although model performance was worse for the week 12 outcomes (see Table 2 and Supplementary Figures 1–4 for the receiver operating characteristic curves).

**Table 2:** Model performance in the test data-set

Time	Outcome	Model	AUC	Accuracy	Sensitivity	Specificity	PPV	NPV
Week 6	Response	Baseline	0.60	0.59	0.67	0.51	0.59	0.59
		Total improvement	0.73	0.70	0.81	0.59	0.67	0.74
		Item improvement	0.77	0.70	0.74	0.66	0.70	0.71
		Item interactions	0.77	0.70	0.72	0.67	0.70	0.70
	Remission	Baseline	0.64	0.68	0.09	0.98	0.69	0.68
		Total improvement	0.74	0.71	0.30	0.92	0.64	0.72
		Item improvement	0.78	0.74	0.44	0.89	0.66	0.76
		Item interactions	0.78	0.74	0.43	0.89	0.66	0.76
Week 12	Response	Baseline	0.62	0.69	1.00	0.00	0.69	N/A
		Total improvement	0.67	0.72	0.93	0.27	0.73	0.62
		Item improvement	0.71	0.71	0.90	0.29	0.73	0.57
		Item interactions	0.70	0.70	0.90	0.29	0.73	0.56
	Remission	Baseline	0.62	0.59	0.61	0.57	0.60	0.58
		Total improvement	0.68	0.64	0.69	0.59	0.64	0.64
		Item improvement	0.74	0.67	0.69	0.64	0.67	0.66
		Item interactions	0.73	0.66	0.65	0.68	0.68	0.65

AUC, area under the (receiver operating characteristic) curve; N/A, not applicable (undefined); NPV, negative predictive value; PPV, positive predictive value.

**Fig. 1:**
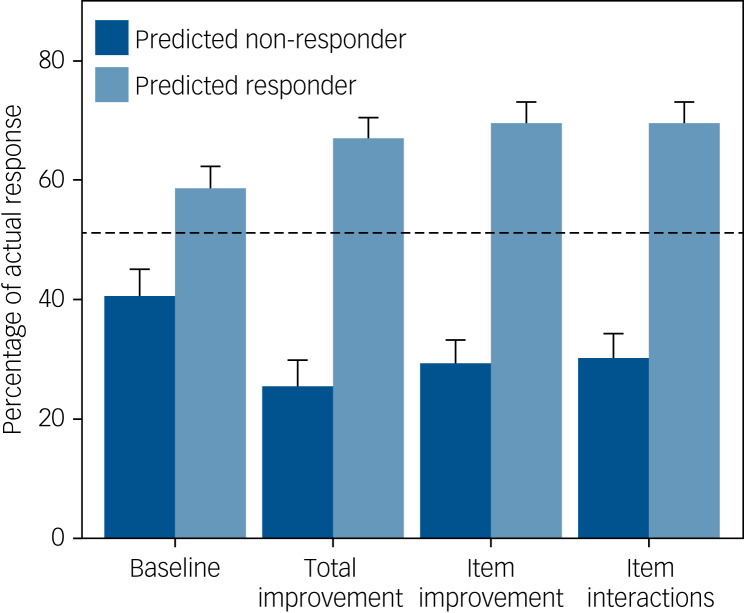
Actual probability of response at week 6 according to participants' predicted outcome (non-response versus response) for each model. Results are based on the test data-set. The dashed line indicates the baseline probability of response. The models predicted non-response for 42% (baseline), 38% (total improvement), 46% (item improvement) and 47% (item interactions) of participants.

The accuracy, sensitivity, specificity, PPV and NPV of each model are also given in Table 2. There were only minor differences between the three early improvement models. At week 6, 51% of antidepressant-treated participants in the test set responded and 33% remitted. The total improvement model predicted non-response for 38% of participants; the associated NPV was 0.74, indicating that 26% of these participants were false negatives who did actually respond by week 6 (Fig. 1). Conversely, of participants who were predicted to respond, 67% actually responded. Based on the most parsimonious model with the highest AUC, the item improvement model, 29% of predicted non-responders actually did respond by week 6 (NPV 0.71), whereas 70% of predicted responders actually responded.

For remission at week 6, the total improvement model identified a large majority group (85% of participants) with a slightly reduced probability of remission (28%) and a minority group with an increased probability of remission (64%). Results were similar for the item improvement model (24% and 66% probability of remission, respectively) (Supplementary Figure 5).

By week 12, 69% of participants in the test set responded and 51% remitted. The total improvement model predicted non-response for 13% of participants, but these participants still had a 38% probability of response, whereas predicted non-responders according to the item improvement model still had a 43% probability of response. For remission, 45% of participants were predicted non-remitters according to the total improvement model, and these had a 36% probability of remission compared with a 34% probability of remission according to the item improvement model (see Supplementary Figures 6 and 7).

*Post hoc*, we also used the predicted probability of responding or remitting to divide participants into quintiles and examined each quintile's actual probability of response or remission (Supplementary Figures 8–11). This more fine-grained approach suggested that the improvement models could identify a risk group with poor outcomes at week 6, but prediction was less accurate and not much better than the baseline model by week 12.

### Secondary analyses

Treatment group (placebo versus SSRI versus SNRI) was a significant predictor of response and remission at both week 6 and week 12. However, models that only included a main effect for treatment group performed as well in the test data-set as models with interactions between group and other variables, indicating no evidence for different associations between early-improving symptoms and response or remission depending on antidepressant class (Supplementary Table 6).

### *Post hoc* analyses

Because the total improvement model performed nearly as well as models that included individual items, we performed additional analyses with continuous change from baseline, as dichotomising a continuous variable might affect model performance. For response at week 6, the lasso regressions for the total improvement, item improvement and item interaction models all selected the same variables (baseline score and change from baseline). The AUC of this model in the test data-set was 0.79. For remission at week 6 and response and remission at week 12, the lasso regressions did select individual items for the item improvement and/or the item interactions model. However, these models had similar AUCs as the (more parsimonious) total improvement model. The AUC for the total improvement model was 0.79 for remission at week 6, 0.71 for response at week 12 and 0.75 for remission at week 12 (Supplementary Table 7).

**Fig. 2:**
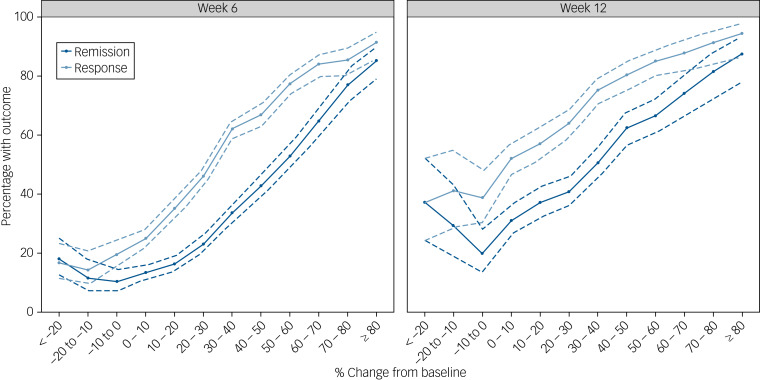
Proportion of participants who responded or remitted according to the percentage improvement from baseline. Error bars (dashed lines) indicate the 95% CIs.

Figure 2 depicts the probability of response or remission as a function of the percentage change from baseline in the total HRSD score at week 2. The probability of response at week 6 was 91% for the few participants (163 out of 6058 participants; 3%) who improved ≥80% by week 2, decreasing to 17% for participants who showed any early worsening (573 participants; 9%). At week 12, however, even participants who showed early worsening still had a 39% probability of responding.

Participants with missing data for week 6 or week 12 outcomes were largely comparable to participants that did have valid data, but were less likely to show early improvement (Supplementary Table 8). In the imputed data-sets, model performance was comparable to our main analyses (Supplementary Table 9), although overall probabilities of response and remission were slightly lower.

## Discussion

### Principal findings

In this individual patient data meta-analysis, we investigated whether early improvement in individual HRSD symptoms could predict response and remission better than early improvement in the total score alone. Consistent with previous literature, we found that patients without early improvement were less likely to respond or remit.^9^^,^^10^ In our main analyses, a model with individual symptoms did perform better than a model that only included total improvement. However, the difference was relatively small, and *post hoc* analyses examining continuous change from baseline did not confirm an added benefit from examining individual symptoms. There was also no evidence that interactions between age, gender and symptoms improved model performance. Our secondary analyses also found no evidence that associations between early-improving symptoms and outcome differed between placebo, SSRIs and SNRIs. Taken together, our results show that early improvement is a non-specific predictor of response and remission, regardless of treatment type.

Although our results confirm the predictive value of early improvement, this value was still relatively limited, especially for longer-term outcomes. Some authors have suggested that non-improvers have virtually no chance of attaining remission and that these patients' treatment should be adapted,^7^ but our results indicate that these patients can still achieve response and remission. Further, although it was possible to identify a small group of participants with a very slim chance of attaining remission by week 6 (7%) by looking specifically at risk quintiles, even participants in the highest-risk quintile still had a 21% chance of remitting and a 48% chance of responding by week 12. This decrease in predictive ability for longer-term outcomes suggests that clinicians can anticipate relatively poor outcomes in early non-improvers by week 6, but that a patient's eventual outcome after 12 weeks of treatment cannot be predicted with any certainty after 2 weeks of treatment.

These findings are consistent with results from another large, 12-week trial (the Genome-Based Therapeutic Drugs for Depression study).^8^ Another study found that the probability that non-responders would respond within the next 2 weeks was stable throughout the first 12 weeks of treatment, at around 15%.^25^ Hence, although lack of early improvement can identify a group of high-risk patients that may need to be monitored more intensively or benefit from additional counselling, a degree of caution in adapting pharmacological treatment may be warranted, especially because the evidence for alternative treatment strategies is scarce. Switching antidepressants is no more effective than continuing the same antidepressant,^26^ and there is also little evidence in favour of the effectiveness of early dose escalation, although escalation is clearly associated with reduced tolerability.^27^^,^^28^ This may, however, be different for non-SSRI/SNRI antidepressants like mirtazapine,^29^ or for escalating from very low dosages to standard dosages.^30^ Other strategies, such as augmentation, may be more successful but also result in decreased tolerability.^31^ Such strategies might be appropriate for some patients without early improvement, for instance if a fast response is essential because of suicidality, but are likely to be premature for many patients. Given the limited predictive accuracy of models based on symptoms alone, inclusion of a broader set of predictors (e.g. psychiatric history, comorbidity or adverse events) may be necessary to achieve better predictions.

Previous research has indicated that symptoms are not interchangeable, and that the depression sum score could obscure important information.^11^ Several studies have also found that early improvement in specific symptoms is associated with good outcomes,^14^^–^^19^ in seeming contrast to our work. However, none of these studies included early improvement in the total score, so their findings are not directly comparable to ours. Furthermore, a variety of symptoms were found to be predictive, including general somatic symptoms, gastrointestinal symptoms, insomnia, depressed mood, agitation, loss of interest, feeling slowed down and others, which also suggests that the association between early-improving symptoms and outcome is not particularly specific. Our lasso regressions also tended to select most of the HRSD items rather than a few specific items, although some items were consistently not selected (suicidality and hypochondriasis). These results suggest that, with regard to early improvement, individual symptoms do not add meaningful predictive information to the sum score (especially when taken as continuous). This may be because symptoms are actually more or less interchangeable in this regard, and early improvement in any symptom is associated with good outcomes, or because symptoms are correlated and tend to improve together. However, it could also be related to the reliability of individual items. Single items are more strongly affected by random error than multi-item scales, for which the random error can balance out, degrading the predictive ability of a symptom. Furthermore, as our outcomes were derived from the HRSD sum score, they are inherently dependent on improvement in all individual items, although this would not *a priori* exclude differences in predictive ability, particularly if the probability or time course of improvement differs.

Our *post hoc* analyses show that the association between early improvement and outcome is gradual. Although a cut-off, such as ≥20% improvement, may be easier to use in clinical practice, there is no major difference between patients on either side of this cut-off value. The likelihood of response or remission does, however, seem to plateau as the percentage improvement by week 2 drops below around 10%. For instance, the likelihood of response by week 6 is only 17% for patients who deteriorate early in treatment, and the likelihood of remission is only 13%. By week 12, however, around 39% of patients who deteriorate early in treatment have responded and 26% have remitted, which suggests that good outcomes are still possible for these patients (although less likely), if a longer period until remission can be tolerated. These results may therefore offer some guidance to clinicians who are faced with patients showing variable degrees of early improvement and need to decide between continuing, switching or intensifying treatment.

### Strengths and limitations

Among the strengths of our study is our large sample size, achieved through combining individual patient data. We used a rigorous approach to building predictive models, including using lasso to prevent over-fitting and using separate test data to examine model performance. We also examined multiple outcomes (response and remission) and both a short and a longer time frame (6 and 12 weeks).

A limitation of our study is that we did not take dosing schedules into account. One study has found that early improvement was more predictive when rapid rather than slow dose escalation was used.^7^ However, there is only limited evidence for a dose-response relationship for SGAs,^30^^,^^32^^,^^33^ and dose escalation usually also continues beyond 2 weeks in clinical practice.

We also took a complete-case approach because we were interested in predicting outcomes in patients who are receiving treatment. Our results therefore do not apply to participants who discontinue their medication and drop out of the trial. However, a sensitivity analysis, in which we imputed missing data for participants who did have a baseline and week 2 visit, but did not have week 6 or week 12 visits, yielded similar model performance, although overall response and remission rates were reduced slightly (e.g. from 51.3% to 49.8% for week 6 response).

An additional limitation is that our data were derived from clinical trials with strict inclusion and exclusion criteria. Hence, the study population represents only a subset of treatment-seeking patients, and participants may, on average, have better outcomes than patients seen in clinical practice.^34^ Further research is therefore necessary to confirm that our results generalise to the broader patient population, including those with extensive comorbidity or chronic depression.

We also specifically examined the ability of early improvement in individual symptoms to predict response or remission. Other clinically relevant outcomes, such as suicidality or functional impairment, were not examined, and it is conceivable that individual symptoms do add predictive information for such outcomes. Similarly, we specifically examined symptoms derived from the clinician-rated HRSD because the HRSD is most commonly used in depression trials. Other instruments, however, may cover a different set of symptoms,^35^ and there could also be differences between self-reported scales and clinician-rated instruments.^36^

Finally, we chose the threshold of ≥50% probability to assign participants to the response or remission category. This is a reasonable cut-off with the advantage of being independent of the data. However, a different cut-off could increase the NPV (at the cost of PPV). In principle, this might identify a group of participants with a lower probability of response or remission. However, because of decreasing specificity, this group would become progressively smaller as NPV increases, which would limit clinical applicability. In *post hoc* analyses, we examined risk quintiles, which suggested that a small group of participants with poor outcomes at week 6 could be identified, but predictive accuracy was reduced for week 12 outcomes. Similar results were obtained when examining continuous early improvement, which suggests that this is the upper bound of predictive accuracy that can be achieved on the basis of symptoms alone.

### Conclusions

Our results show that a model with only early improvement in the total score is about as predictive as models that also contain individual symptoms. Hence, clinicians need not focus on specific symptoms, but can gain as much information about the likelihood of response or remission from improvement in the total score alone, particularly if improvement is interpreted as a continuous measure. However, although absence of early improvement identifies a group of patients that is at increased risk of poor outcomes and that may need to be monitored more closely, it does not rule out later response or remission with certainty. Therefore, adapting antidepressant treatment because of limited improvement in the first 2 weeks would be premature for many patients.

## Supplementary material

For supplementary material accompanying this paper visit http://dx.doi.org/10.1192/bjp.2018.122. click here to view supplementary material

## References

[1] National Institute for Health and Care Excellence. *Depression: The NICE Guideline on the Treatment and Management of Depression in Adults (CG90)* NICE, 2010.

[2] Spijker J, Bockting CLH, Meeuwissen JAC, van Vliet IM, Emmelkamp PMG, Hermens MLM, Multidisciplinaire richtlijn Depressie (Derde revisie) : Richtlijn voor de diagnostiek, behandeling en begeleiding van volwassen patiënten met een depressieve stoornis. Trimbos-Instituut, 2013.

[3] American Psychiatric Association. *Practice Guideline for the Treatment of Patients with Major Depressive Disorder* American Psychiatric Association, 2010.

[4] Undurraga J, Baldessarini RJ. Randomized, placebo-controlled trials of antidepressants for acute major depression: thirty-year meta-analytic review. Neuropsychopharmacology 2012; 37: 851–64. 22169941 10.1038/npp.2011.306 PMC3280655

[5] Taylor MJ, Freemantle N, Geddes JR, Bhagwagar Z. Early onset of selective serotonin reuptake inhibitor antidepressant action: a systematic review and meta-analysis. Arch Gen Psychiatry 2006; 63: 1217–23. 17088502 10.1001/archpsyc.63.11.1217 PMC2211759

[6] Stassen HH, Angst J, Hell D, Scharfetter C, Szegedi A. Is there a common resilience mechanism underlying antidepressant drug response? Evidence from 2848 patients. J Clin Psychiatry 2007; 68: 1195–205. 17854243 10.4088/jcp.v68n0805

[7] Szegedi A, Jansen WT, Van Willigenburg APP, Van Der Meulen E, Stassen HH, Thase ME. Early improvement in the first 2 weeks as a predictor of treatment outcome in patients with major depressive disorder: a meta-analysis including 6562 patients. J Clin Psychiatry 2009; 70: 344–53. 19254516 10.4088/jcp.07m03780

[8] Uher R, Mors O, Rietschel M, Rajewska-Rager A, Petrovic A, Zobel A, Early and delayed onset of response to antidepressants in individual trajectories of change during treatment of major depression: a secondary analysis of data from the Genome-Based Therapeutic Drugs for Depression (GENDEP) study. J Clin Psychiatry 2011; 72: 1478–84. 22127194 10.4088/JCP.10m06419

[9] Gorwood P, Bayle F, Vaiva G, Courtet P, Corruble E, Llorca P-M. Is it worth assessing progress as early as week 2 to adapt antidepressive treatment strategy? Results from a study on agomelatine and a global meta-analysis. Eur Psychiatry 2013; 28: 362–71. 23416024 10.1016/j.eurpsy.2012.11.004

[10] Wagner S, Engel A, Engelmann J, Herzog D, Dreimüller N, Müller MB, Early improvement as a resilience signal predicting later remission to antidepressant treatment in patients with major depressive disorder: systematic review and meta-analysis. J Psychiatr Res 2017; 94: 96–106. 28697423 10.1016/j.jpsychires.2017.07.003

[11] Fried EI, Nesse RM. Depression sum-scores don't add up: why analyzing specific depression symptoms is essential. BMC Med 2015; 13: 72. 25879936 10.1186/s12916-015-0325-4 PMC4386095

[12] Uher R, Perlis RH, Henigsberg N, Zobel A, Rietschel M, Mors O, Depression symptom dimensions as predictors of antidepressant treatment outcome: replicable evidence for interest-activity symptoms. Psychol Med 2012; 42: 967–80. 21929846 10.1017/S0033291711001905 PMC3787526

[13] Chekroud AM, Zotti RJ, Shehzad Z, Gueorguieva R, Johnson MK, Trivedi MH, Cross-trial prediction of treatment outcome in depression: a machine learning approach. Lancet Psychiatry 2016; 3: 243–50. 26803397 10.1016/S2215-0366(15)00471-X

[14] Tokuoka H, Takahashi H, Ozeki A, Kuga A, Yoshikawa A, Tsuji T, Trajectories of depression symptom improvement and associated predictor analysis: an analysis of duloxetine in double-blind placebo-controlled trials. J Affect Disord 2016; 196: 171–80. 26922146 10.1016/j.jad.2016.02.039

[15] Funaki K, Nakajima S, Suzuki T, Mimura M, Uchida H. Early improvements in individual symptoms to predict later remission in major depressive disorder treated with mirtazapine. J Clin Pharmacol 2016; 56: 1111–9. 26813241 10.1002/jcph.710

[16] Sakurai H, Uchida H, Abe T, Nakajima S, Suzuki T, Pollock BG, Trajectories of individual symptoms in remitters versus non-remitters with depression. J Affect Disord 2013; 151: 506–13. 23886402 10.1016/j.jad.2013.06.035

[17] Mizushima J, Uchida H, Tada M, Suzuki T, Mimura M, Nio S. Early improvement of specific symptoms predicts subsequent recovery in bipolar depression. J Clin Psychiatry 2017; 78: e146–51. 28234437 10.4088/JCP.15m10573

[18] Berlin I, Lavergne F. Early predictors of two month response with mianserin and selective serotonin reuptake inhibitors and influence of definition of outcome on prediction. Eur Psychiatry 1998; 13: 138–42. 19698616 10.1016/S0924-9338(98)80137-5

[19] Farabaugh AH, Bitran S, Witte J, Alpert J, Chuzi S, Clain AJ, Anxious depression and early changes in the HAMD-17 anxiety-somatization factor items and antidepressant treatment outcome. Int Clin Psychopharmacol 2010; 25: 214–7. 20400905 10.1097/YIC.0b013e328339fbbd PMC2909033

[20] Clinical Study Data Request (CSDR). ClinicalStudyDataRequest.com. CSDR, 2018 (https://clinicalstudydatarequest.com/).

[21] Leucht S, Fennema H, Engel R, Kaspers-Janssen M, Lepping P, Szegedi A. What does the HAMD mean? J Affect Disord 2013; 148: 243–8. 23357658 10.1016/j.jad.2012.12.001

[22] Cipriani A, Furukawa TA, Salanti G, Chaimani A, Atkinson LZ, Ogawa Y, Comparative efficacy and acceptability of 21 antidepressant drugs for the acute treatment of adults with major depressive disorder: a systematic review and network meta-analysis. Lancet 2018; 391(10128): 1357–66. 29477251 10.1016/S0140-6736(17)32802-7 PMC5889788

[23] Tibshirani R. Regression selection and shrinkage via the lasso. J R Stat Soc B 1996; 58: 267–88.

[24] Friedman J, Hastie T, Tibshirani R. Regularization paths for generalized linear models via coordinate descent. J Stat Softw 2010; 33: 1–22. 20808728 PMC2929880

[25] Posternak MA, Baer L, Nierenberg AA, Fava M. Response rates to fluoxetine in subjects who initially show no improvement. J Clin Psychiatry 2011; 72: 949–54. 21672502 10.4088/JCP.10m06098

[26] Bschor T, Kern H, Henssler J, Baethge C. Switching the antidepressant after nonresponse in adults with major depression: a systematic literature search and meta-analysis. J Clin Psychiatry 2018; 79(1): 16r10749. 10.4088/JCP.16r10749 27929611

[27] Ruhé HG, Huyser J, Swinkels JA, Schene AH. Dose escalation for insufficient response to standard-dose selective serotonin reuptake inhibitors in major depressive disorder - systematic review. Br J Psychiatry 2006; 189: 309–16. 17012653 10.1192/bjp.bp.105.018325

[28] Dold M, Bartova L, Rupprecht R, Kasper S. Dose escalation of antidepressants in unipolar depression: a meta-analysis of double-blind, randomized controlled trials. Psychother Psychosom 2017; 86: 283–91. 28903107 10.1159/000477770

[29] Ueno F, Nakajima S, Suzuki T, Abe T, Sato Y, Mimura M, Whether to increase or maintain dosage of mirtazapine in early nonimprovers with depression. J Clin Psychiatry 2015; 76: 434–9. 25919835 10.4088/JCP.14m09201

[30] Hieronymus F, Nilsson S, Eriksson E. A mega-analysis of fixed-dose trials reveals dose-dependency and a rapid onset of action for the antidepressant effect of three selective serotonin reuptake inhibitors. Transl Psychiatry 2016; 6: e834. 27271860 10.1038/tp.2016.104 PMC4931602

[31] Zhou X, Ravindran A V, Qin B, Del Giovane C, Li Q, Bauer M, Comparative efficacy, acceptability, and tolerability of augmentation agents in treatment-resistant depression. J Clin Psychiatry 2015; 76: e487–98. 25919841 10.4088/JCP.14r09204

[32] Adli M, Baethge C, Heinz A, Langlitz N, Bauer M. Is dose escalation of antidepressants a rational strategy after a medium-dose treatment has failed? A systematic review. Eur Arch Psychiatry Clin Neurosci 2005; 255: 387–400. 15868067 10.1007/s00406-005-0579-5

[33] Jakubovski E, Varigonda AL, Freemantle N, Taylor MJ, Bloch MH. Systematic review and meta-analysis: dose-response relationship of selective serotonin reuptake inhibitors in major depressive disorder. Am J Psychiatry 2015; 54: 557–64. 10.1016/j.jaac.2015.05.004 26088660

[34] Wisniewski SR, Rush AJ, Nierenberg AA, Gaynes BN, Warden D, Luther JF, Can phase III trial results of antidepressant medications be generalized to clinical practice? A STAR*D report. Am J Psychiatry 2009; 166: 599–607. 19339358 10.1176/appi.ajp.2008.08071027

[35] Gullion CM, Rush AJ. Toward a generalizable model of symptoms in major depressive disorder. Biol Psychiatry 1998; 44: 959–72. 9821560 10.1016/s0006-3223(98)00235-2

[36] Uher R, Perlis RH, Placentino A, Dernovšek MZ, Henigsberg N, Mors O, Self-report and clinician-rated measures of depression severity: can one replace the other? Depress Anxiety 2012; 29: 1043–9. 22933451 10.1002/da.21993 PMC3750710

