# Supplementary material for: Predicting antidepressant response by monitoring early improvement of individual symptoms of depression: individual patient data meta-analysis
Source: Br J Psychiatry. 2018 Jun 28;214(1):4–10. doi: 10.1192/bjp.2018.122 (PMC7557872; doi:10.1192/bjp.2018.122)
Supplement: Supplementary file 1 [file S0007125018001228sup001.docx]

**Supplemental table 1. Table of study characteristics.**

| **Drug** | **Trial** | **Ref.** | **Duration (weeks)** | **Antidepressant dose (mg/day)** | **Sample size (week 6 analyses)** | | **Mean baseline score (SD)** | **Mean age in years (SD)** | **Gender (% female)** |
| --- | --- | --- | --- | --- | --- | --- | --- | --- | --- |
|  |  |  |  |  | **Placebo** | **Drug** |  |  |  |
| Paroxetine (immediate release) | 01/001 | (1) | 6 | 10 – 50 | 18 | 19 | 25.0 (3.2) | 42.8 (12.3) | 35.1 |
|  | 02/001 - 004 | (2–5) | 6 | 10 – 50 | 100 | 112 | 23.5 (4.0) | 41.5 (12.2) | 53.3 |
|  | 03/001 - 006 | (6) | 6 | 10 – 50 | 117 | 141 | 23.3 (3.7) | 41.3 (11.7) | 49.6 |
|  | 07 | (7) | 6 | 10 – 60 | 7 | 8 | 25.1 ( 4.2) | 41.0 (7.5) | 53.3 |
|  | 09 | (8) | 6 | 10, 20, 30, 40 | 31 | 262 | 22.5 (3.0) | 41.4 (12.5) | 54.3 |
|  | 115 | (9) | 12 | 20  Fluoxetine (20) | 92 | 206  215 | 22.3 (3.6) | 42.4 (11.8) | 66.1 |
|  | 128 | (10) | 12 | 20  Fluoxetine (20) | 115 | 257  276 | 23.1 (3.8) | 41.2 (11.7) | 63.9 |
|  | 276 | (11) | 6 | 30 | 13 | 15 | 22.8 (3.9) | 42.0 (13.1) | 46.4 |
|  | 279 | (12) | 6 | 30 | 7 | 14 | 20.8 (3.7) | 42.1 (18.9) | 71.4 |
| Paroxetine (controlled release) | 448 | (13) | 12 | IR 20 – 50  CR 25 – 62.5 | 94 | 173 | 23.3 (2.8) | 39.0 (10.2) | 61.4 |
|  | 449 | (13) | 12 | IR 20 – 50  CR 25 – 62.5 | 97 | 193 | 23.6 (3.1) | 41.0 (11.4) | 67.2 |
|  | 487 | (14) | 12 | IR 10 – 40  CR 12.5 – 50 | 97 | 189 | 22.1 (3.1) | 69.8 (5.9) | 55.9 |
|  | 810 | (15) | 8 | 12.5, 25 | 128 | 267 | 23.5 (3.1) | 39.4 (11.6) | 58.7 |
| Duloxetine | HMAQ-A | (16) | 8 | 40 – 120  Fluoxetine 20 | 56 | 56  27 | 18.5 (4.4) | 41.3 (11.6) | 65.8 |
|  | HMAQ-B | (17) | 8 | 40 – 120  Fluoxetine 20 | 60 | 67  29 | 18.1 (5.2) | 41.1 (11.1) | 68.0 |
|  | HMAT-A | (18) | 8 | 40, 80  Paroxetine 20 | 76 | 142  73 | 17.5 (5.3) | 43.6 (14.3) | 60.1 |
|  | HMAT-B | (19) | 8 | 40, 80  Paroxetine 20 | 71 | 142  66 | 17.9 (5.2) | 40.3 (11.1) | 60.2 |
|  | HMAY-A | (20) | 8 | 80, 120  Paroxetine 20 | 87 | 171  79 | 20.0 (3.7) | 43.4 (11.0) | 71.5 |
|  | HMAY-B | (21) | 8 | 80, 120  Paroxetine 20 | 96 | 184  89 | 21.0 (3.6) | 45.1 (11.0) | 69.9 |
|  | HMBH-A | (22) | 9 | 60 | 102 | 99 | 21.2 (4.0) | 42.7 (13.4) | 67.7 |
|  | HMBH-B | (23) | 9 | 60 | 112 | 99 | 20.5 (3.4) | 42.3 (13.6) | 67.8 |
|  | HMBU | (24) | 12 | 60 - 120  Venlafaxine 75 - 225 | - | 137  153 | 23.1 (3.7) | 43.9 (13.2) | 70.0 |
|  | HMCQ | (24) | 12 | 60 - 120  Venlafaxine 75 - 225 | - | 133  294 | 22.3 (3.3) | 42.4 (11.9) | 63.2 |
|  | HMCR | (25) | 8 | 60  Escitalopram 10 | 113 | 225  237 | 17.7 (5.0) | 43.1 (12.1) | 65.9 |
|  | HMCV | (26) | 8 | 60  Paroxetine 20 | - | 183  204 | 21.2 (3.9) | 37.7 (13.8) | 68.2 |
|  | HMFA | (27) | 12 | 60 | 89 | 205 | 18.8 (6.3) | 72.5 (5.8) | 64.3 |
|  | HMFS-A | (28) | 12 | 60 | 103 | 221 | 22.9 (4.2) | 43.0 (12.3) | 63.0 |
|  | HMFS-B | (28) | 12 | 60 | 111 | 225 | 22.8 (4.7) | 45.0 (12.0) | 64.9 |
| New chemical entities | NKD20006 | (29) | 8 | Paroxetine 20 | 95 | 83 | 24.5 (2.8) | 38.0 (11.8) | 58.4 |
|  | NKF100096 | (30) | 8 | Paroxetine 20 - 30 | 97 | 88 | 22.2 (5.6) | 43.9 (10.8) | 73.5 |

**References**

1. GSK Clinical Trial Registry: A phase II, placebo-controlled, double-blind study of paroxetine in depressed outpatients [Internet]. Available from: https://www.gsk-clinicalstudyregister.com/study/29060/01/001

2. Claghorn J: A double-blind comparison of paroxetine and placebo in the treatment of depressed outpatients. Int. Clin. Psychopharmacol. 1992; 6 Suppl 4:25–30

3. Kiev A: A double-blind, placebo-controlled study of paroxetine in depressed outpatients. J. Clin. Psychiatry 1992; 53:27–29

4. Rickels K, Amsterdam J, Clary C, Fox I, Schweizer E, Weise C: A placebo-controlled, double-blind, clinical trial of paroxetine in depressed outpatients. Acta Psychiatr. Scand. Suppl. 1989; 80:117–123

5. Smith WT, Glaudin V: A placebo-controlled trial of paroxetine in the treatment of major depression. J. Clin. Psychiatry 1992; 53:36–39

6. Feighner JP, Cohn JB, Fabre LF, Fieve RR, Mendels J, Shrivastava RK, Dunbar GC: A study comparing paroxetine placebo and imipramine in depressed patients. J. Affect. Disord. 1993; 28:71–9

7. GSK Clinical Trial Registry: A double-blind comparison of paroxetine, amitriptyline, and placebo in inpatients with major depressive disorder with melancholia [Internet]. Available from: https://www.gsk-clinicalstudyregister.com/study/29060/07/001

8. GSK Clinical Trial Registry: A multicenter, double-blind, placebo-controlled fixed-dose evaluation of four doses of paroxetine [Internet]. Available from: https://www.gsk-clinicalstudyregister.com/study/29060/009

9. GSK Clinical Trial Registry: A multicenter, randomized, double-blind, placebo-controlled comparison of paroxetine and fluoxetine in the treatment of major depressive disorder [Internet]. Available from: https://www.gsk-clinicalstudyregister.com/study/29060/115

10. GSK Clinical Trial Registry: A multicenter, randomized, double-blind, placebo-controlled comparison of paroxetine and fluoxetine in the treatment of major depressive disorder [Internet]. Available from: https://www.gsk-clinicalstudyregister.com/study/29060/128

11. Edwards JG, Goldie A: Placebo-controlled trial of paroxetine in depressive illness. Hum. Psychopharmacol. 1993; 8:203–209

12. GSK Clinical Trial Registry: A study to assess the effectiveness and tolerance of paroxetine by double-blind comparison with placebo and mianserin [Internet]. Available from: https://www.gsk-clinicalstudyregister.com/study/29060/012_3

13. Golden RN, Nemeroff CB, McSorley P, Pitts CD, Dubé EM: Efficacy and tolerability of controlled-release and immediate-release paroxetine in the treatment of depression. J. Clin. Psychiatry 2002; 63:577–84

14. Rapaport MH, Schneider LS, Dunner DL, Davies JT, Pitts CD: Efficacy of controlled-release paroxetine in the treatment of late-life depression. J. Clin. Psychiatry 2003; 64:1065–1074

15. Trivedi MH, Pigotti TA, Perera P, Dillingham KE, Carfagno ML, Pitts CD: Effectiveness of low doses of paroxetine controlled release in the treatment of major depressive disorder. J. Clin. Psychiatry 2004; 65:1356–64

16. Goldstein DJ, Mallinckrodt C, Lu Y, Demitrack MA: Duloxetine in the treatment of major depressive disorder: A double-blind clinical trial. J. Clin. Psychiatry 2002; 63:225–231

17. Lilly clinical trial registry: Duloxetine versus placebo in the treatment of major depression [Internet]. Available from: https://assets.contentful.com/hadumfdtzsru/5FZ15aVRBKKsmKIoeSgsMO/b1b988aefbb263aabdb413af7f3534a7/Duloxetine-F1J-MC-HMAQ-_Study-Group-B_.pdf

18. Lilly clinical trial registry: Duloxetine versus placebo and paroxetine in the acute treatment of major depression, study group A [Internet]. 2004; Available from: https://assets.contentful.com/hadumfdtzsru/1D76MTikKkeSIuOS4SgQcq/433330f08ad190a430f5d0f2140cb4b3/Duloxetine-F1J-MC-HMAT-_Study-Group-A_.pdf

19. Goldstein DJ, Lu Y, Detke MJ, Wiltse C, Mallinckrodt C, Demitrack MA: Duloxetine in the treatment of depression: a double-blind placebo-controlled comparison with paroxetine. J. Clin. Psychopharmacol. 2004; 24:389–399

20. Detke MJ, Wiltse CG, Mallinckrodt CH, McNamara RK, Demitrack MA, Bitter I: Duloxetine in the acute and long-term treatment of major depressive disorder: A placebo- and paroxetine-controlled trial. Eur. Neuropsychopharmacol. 2004; 14:457–470

21. Perahia DGS, Kajdasz DK, Royer MG, Walker DJ, Raskin J: Duloxetine in the treatment of major depressive disorder: an assessment of the relationship between outcomes and episode characteristics. Int. Clin. Psychopharmacol. 2006; 21:285–95

22. Detke MJ, Lu Y, Goldstein DJ, Hayes JR, Demitrack MA: Duloxetine, 60 mg once daily, for major depressive disorder: A randomized double-blind placebo-controlled trial. J. Clin. Psychiatry 2002; 63:308–315

23. Detke MJ, Lu Y, Goldstein DJ, McNamara RK, Demitrack MA: Duloxetine 60 mg once daily dosing versus placebo in the acute treatment of major depression. J. Psychiatr. Res. 2002; 36:383–390

24. Perahia DGS, Pritchett YL, Kajdasz DK, Bauer M, Jain R, Russell JM, Walker DJ, Spencer KA, Froud DM, Raskin J, Thase ME: A randomized, double-blind comparison of duloxetine and venlafaxine in the treatment of patients with major depressive disorder. J. Psychiatr. Res. 2008; 42:22–34

25. Nierenberg AA, Greist JH, Mallinckrodt CH, Prakash A, Sambunaris A, Tollefson GD, Wohlreich MM: Duloxetine versus escitalopram and placebo in the treatment of patients with major depressive disorder: onset of antidepressant action, a non-inferiority study. Curr. Med. Res. Opin. 2007; 23:401–416

26. Lee P, Shu L, Xu X, Wang CY, Lee MS, Liu CY, Hong JP, Ruschel S, Raskin J, Colman SA, Harrison GA: Once-daily duloxetine 60 mg in the treatment of major depressive disorder: Multicenter, double-blind, randomized, paroxetine-controlled, non-inferiority trial in China, Korea, Taiwan and Brazil. Psychiatry Clin. Neurosci. 2007; 61:295–307

27. Oakes TM, Katona C, Liu P, Robinson M, Raskin J, Greist JH: Safety and tolerability of duloxetine in elderly patients with major depressive disorder: a pooled analysis of two placebo-controlled studies. Int. Clin. Psychopharmacol. 2013; 28:1–11

28. Oakes TMM, Myers AL, Marangell LB, Ahl J, Prakash A, Thase ME, Kornstein SG: Assessment of depressive symptoms and functional outcomes in patients with major depressive disorder treated with duloxetine versus placebo: Primary outcomes from two trials conducted under the same protocol. Hum. Psychopharmacol. 2012; 27:47–56

29. GSK Clinical Trial Registry: An 8-week, randomized, double-blind, placebo-controlled, multicenter, fixed-dose study comparing the efficacy and safety of a new chemical entity (NCE) or paroxetine to placebo in moderately to severely depressed patients with major depressive disorder [Internet]. Available from: https://www.gsk-clinicalstudyregister.com/study/NKD20006

30. GSK Clinical Trial Registry: A randomised, double-blind, double-dummy, parallel-group, placebo-controlled, forced dose titration study evaluating the efficacy and safety of a new chemical entity (NCE) and paroxetine in subjects with major depressive disorder [Internet]. Available from: https://www.gsk-clinicalstudyregister.com/study/NKF100096

**Supplemental table 2. Model coefficients for response at week 6.**

|  | **Baseline** | **Total improvement** | **Item improvement** | **Item interactions** |
| --- | --- | --- | --- | --- |
| Constant | -0.874 (0.457) | -0.952 (0.082) | -1.680 (0.100) | -1.271 (0.124) |
| Age | -0.059 (0.034) |  |  |  |
| HAM-D item 1 | 0.736 (0.452) |  |  |  |
| HAM-D item 6 | 0.135 (0.066) |  |  |  |
| HAM-D item 9 | 0.052 (0.063) |  |  |  |
| HAM-D item 12 | 0.058 (0.063) |  |  |  |
| HAM-D item 16 | 0.107 (0.077) |  |  |  |
| HAM-D item 17 | 0.157 (0.082) |  |  |  |
| Total improvement |  | 1.606 (0.067) | 0.600 (0.094) | 0.734 (0.099) |
| Improv item 1 |  |  | 0.351 (0.077) | 0.326 (0.138) |
| Improv item 2 |  |  | 0.376 (0.069) | 0.077 (0.146) |
| Improv item 4 |  |  | 0.182 (0.069) | -0.122 (0.112) |
| Improv item 5 |  |  | 0.302 (0.070) | 0.219 (0.143) |
| Improv item 6 |  |  | 0.243 (0.070) | 0.119 (0.093) |
| Improv item 7 |  |  | 0.197 (0.072) | -0.128 (0.125) |
| Improv item 8 |  |  | 0.148 (0.070) | -0.094 (0.117) |
| Improv item 9 |  |  | 0.220 (0.069) | 0.172 (0.127) |
| Improv item 10 |  |  | 0.274 (0.070) | 0.091 (0.149) |
| Improv item 11 |  |  | 0.231 (0.068) | -0.029 (0.134) |
| Improv item 12 |  |  | 0.195 (0.079) | 0.066 (0.103) |
| Improv item 13 |  |  | 0.328 (0.073) | -0.044 (0.148) |
| Improv item 14 |  |  | 0.319 (0.081) | -0.013 (0.174) |
| Improv item 16 |  |  |  | -0.120 (0.113) |
| Improv 1 * improv 2 |  |  |  | 0.034 (0.149) |
| Improv 1 * improv 5 |  |  |  | 0.014 (0.146) |
| Improv 1 * improv 9 |  |  |  | -0.015 (0.144) |
| Improv 1 * improv 10 |  |  |  | -0.025 (0.150) |
| Improv 1 * improv 14 |  |  |  | 0.163 (0.184) |
| Improv 2 * improv 8 |  |  |  | 0.220 (0.140) |
| Improv 2 * improv 10 |  |  |  | 0.075 (0.139) |
| Improv 2 * improv 11 |  |  |  | 0.202 (0.137) |
| Improv 2 * improv 13 |  |  |  | 0.173 (0.146) |
| Improv 4 * improv 7 |  |  |  | 0.333 (0.137) |
| Improv 5 * improv 9 |  |  |  | 0.130 (0.141) |
| Improv 5 * improv 10 |  |  |  | -0.015 (0.140) |
| Improv 5 * improv 11 |  |  |  | 0.037 (0.141) |
| Improv 6 * improv 8 |  |  |  | 0.192 (0.142) |
| Improv 6 * improv 14 |  |  |  | 0.255 (0.171) |
| Improv 8 * improv 12 |  |  |  | 0.232 (0.163) |
| Improv 10 * improv 7 |  |  |  | 0.240 (0.142) |
| Improv 10 * improv 11 |  |  |  | 0.067 (0.138) |
| Improv 10 * improv 13 |  |  |  | 0.055 (0.149) |
| Improv 11 * improv 4 |  |  |  | 0.268 (0.141) |
| Improv 13 * improv 7 |  |  |  | 0.142 (0.148) |
| Improv 13 * improv 16 |  |  |  | 0.788 (0.213) |
| Improv 14 * improv 13 |  |  |  | 0.217 (0.170) |
| Observations | 4,847 | 4,847 | 4,847 | 4,847 |
| Log Likelihood | -3,291.889 | -2,982.251 | -2,864.412 | -2,834.873 |
| AIC | 6,601.778 | 5,970.503 | 5,760.824 | 5,749.746 |
| BIC | 6,660.153 | 5,989.961 | 5,864.602 | 6,009.191 |

**Supplemental table 3. Model coefficients for remission at week 6.**

|  | **Baseline** | **Total improvement** | **Item improvement** | **Item interactions** |
| --- | --- | --- | --- | --- |
| Constant | -0.775 (0.071) | -1.880 (0.088) | -2.610 (0.229) | -2.170 (0.149) |
| Baseline score | -0.424 (0.037) | -0.493 (0.039) | -0.709 (0.047) | -0.732 (0.044) |
| HAM-D item 2 |  |  | -0.167 (0.121) |  |
| HAM-D item 12 |  |  | 0.160 (0.091) |  |
| HAM-D item 13 |  |  | -0.241 (0.165) |  |
| HAM-D item 16 |  |  | 0.139 (0.166) |  |
| Total improvement |  | 1.591 (0.080) | 0.395 (0.110) | 0.692 (0.121) |
| Improv item 1 |  |  | 0.390 (0.088) | 0.218 (0.170) |
| Improv item 2 |  |  | 0.539 (0.081) | 0.107 (0.182) |
| Improv item 4 |  |  | 0.238 (0.073) | -0.127 (0.161) |
| Improv item 5 |  |  | 0.321 (0.074) | 0.108 (0.171) |
| Improv item 6 |  |  | 0.205 (0.075) | -0.055 (0.170) |
| Improv item 7 |  |  | 0.283 (0.077) | -0.179 (0.181) |
| Improv item 8 |  |  | 0.274 (0.074) | -0.031 (0.142) |
| Improv item 9 |  |  | 0.192 (0.073) | -0.232 (0.149) |
| Improv item 10 |  |  | 0.274 (0.076) | -0.177 (0.161) |
| Improv item 11 |  |  | 0.251 (0.073) | 0.046 (0.137) |
| Improv item 12 |  |  | 0.097 (0.099) | 0.056 (0.107) |
| Improv item 13 |  |  | 0.442 (0.076) | 0.008 (0.168) |
| Improv item 14 |  |  | 0.433 (0.081) | -0.129 (0.194) |
| Improv item 16 |  |  | 0.250 (0.181) | -0.007 (0.216) |
| Improv item 17 |  |  | 0.274 (0.125) | 0.086 (0.140) |
| Improv 1 * improv 2 |  |  |  | -0.197 (0.172) |
| Improv 1 * improv 4 |  |  |  | 0.147 (0.177) |
| Improv 1 * improv 5 |  |  |  | 0.106 (0.175) |
| Improv 1 * improv 6 |  |  |  | -0.022 (0.175) |
| Improv 1 * improv 7 |  |  |  | 0.222 (0.173) |
| Improv 1 * improv 14 |  |  |  | 0.272 (0.203) |
| Improv 1 * improv 16 |  |  |  | 0.167 (0.232) |
| Improv 2 * improv 6 |  |  |  | 0.195 (0.149) |
| Improv 2 * improv 7 |  |  |  | 0.107 (0.158) |
| Improv 2 * improv 8 |  |  |  | 0.256 (0.150) |
| Improv 2 * improv 9 |  |  |  | 0.225 (0.150) |
| Improv 2 * improv 10 |  |  |  | 0.208 (0.153) |
| Improv 2 * improv 11 |  |  |  | 0.083 (0.147) |
| Improv 2 * improv 13 |  |  |  | 0.015 (0.154) |
| Improv 4 * improv 7 |  |  |  | 0.243 (0.153) |
| Improv 4 * improv 8 |  |  |  | 0.190 (0.150) |
| Improv 5 * improv 10 |  |  |  | 0.048 (0.151) |
| Improv 5 * improv 11 |  |  |  | 0.144 (0.145) |
| Improv 5 * improv 13 |  |  |  | 0.027 (0.149) |
| Improv 6 * improv 8 |  |  |  | 0.102 (0.149) |
| Improv 6 * improv 9 |  |  |  | 0.277 (0.147) |
| Improv 7 * improv 10 |  |  |  | 0.203 (0.155) |
| Improv 9 * improv 10 |  |  |  | 0.288 (0.154) |
| Improv 10 * improv 13 |  |  |  | 0.215 (0.156) |
| Improv 11 * improv 13 |  |  |  | 0.201 (0.147) |
| Improv 14 * improv 12 |  |  |  | 0.250 (0.184) |
| Improv 14 * improv 13 |  |  |  | 0.491 (0.167) |
| Improv 16 * improv 8 |  |  |  | 0.233 (0.203) |
| Improv 16 * improv 12 |  |  |  | 0.225 (0.210) |
| Improv 16 * improv 17 |  |  |  | 1.010 (0.334) |
| Observations | 4,847 | 4,847 | 4,847 | 4,847 |
| Log Likelihood | -2,947.784 | -2,712.485 | -2,547.361 | -2,512.073 |
| AIC | 5,901.569 | 5,432.971 | 5,140.722 | 5,122.146 |
| BIC | 5,921.027 | 5,458.915 | 5,289.903 | 5,439.966 |

**Supplemental table 4. Model coefficients for response at week 12.**

|  | **Baseline** | **Total improvement** | **Item improvement** | **Item interactions** |
| --- | --- | --- | --- | --- |
| Constant | 0.773 (0.140) | 0.081 (0.130) | 0.007 (0.161) | -0.051 (0.155) |
| Age |  | -0.228 (0.065) | -0.235 (0.066) | -0.222 (0.066) |
| HAM-D item 4 |  |  | -0.580 (0.134) |  |
| Total improvement |  | 1.202 (0.104) | 0.394 (0.146) | 0.452 (0.149) |
| Improv item 1 |  |  | 0.456 (0.126) | 0.475 (0.163) |
| Improv item 2 |  |  | 0.306 (0.116) | 0.010 (0.173) |
| Improv item 4 |  |  | 0.536 (0.129) | 0.237 (0.201) |
| Improv item 7 |  |  | 0.168 (0.120) | 0.119 (0.143) |
| Improv item 6 |  |  |  | -0.416 (0.176) |
| Improv item 10 |  |  | 0.293 (0.116) | -0.331 (0.180) |
| Improv item 12 |  |  |  | -0.285 (0.185) |
| Improv item 13 |  |  | 0.411 (0.124) | -0.046 (0.274) |
| Improv 1 * improv 4 |  |  |  | -0.028 (0.239) |
| Improv 1 * improv 13 |  |  |  | -0.042 (0.274) |
| Improv 2 * improv 10 |  |  |  | 0.505 (0.222) |
| Improv 4 * improv 13 |  |  |  | 0.195 (0.247) |
| Improv 10 * improv 6 |  |  |  | 0.675 (0.231) |
| Improv 10 * improv 12 |  |  |  | 0.793 (0.272) |
| Improv 13 * improv 2 |  |  |  | 0.138 (0.248) |
| Improv 13 * improv 6 |  |  |  | 0.494 (0.247) |
| Improv 13 * improv 7 |  |  |  | 0.161 (0.254) |
| Improv 13 * improv 10 |  |  |  | 0.127 (0.245) |
| Observations | 1,961 | 1,961 | 1,961 | 1,961 |
| Log Likelihood | -1,200.156 | -1,125.716 | -1,085.160 | -1,076.193 |
| AIC | 2,404.312 | 2,259.431 | 2,192.319 | 2,196.386 |
| BIC | 2,415.474 | 2,281.756 | 2,253.712 | 2,319.172 |

**Supplemental table 5. Model coefficients for remission at week 12.**

|  | **Baseline** | **Total improvement** | **Item improvement** | **Item interactions** |
| --- | --- | --- | --- | --- |
| Constant | 0.305 (0.150) | -0.425 (0.160) | -1.034 (0.183) | -0.552 (0.226) |
| Age | -0.135 (0.059) | -0.142 (0.059) | -0.139 (0.061) | -0.065 (0.079) |
| Gender |  |  |  | -0.063 (0.149) |
| Baseline score | -0.270 (0.056) | -0.328 (0.058) | -0.398 (0.062) | -0.436 (0.066) |
| HAM-D item 3 | -0.298 (0.102) | -0.231 (0.106) | -0.173 (0.109) | -0.160 (0.113) |
| HAM-D item 4 | -0.322 (0.109) | -0.395 (0.113) | -0.588 (0.134) | -0.593 (0.138) |
| HAM-D item 16 | 0.355 (0.121) | 0.361 (0.124) | 0.430 (0.129) | 0.474 (0.222) |
| Total improvement |  | 1.151 (0.103) | 0.096 (0.148) | 0.261 (0.163) |
| Improv item 1 |  |  | 0.557 (0.125) | 0.487 (0.207) |
| Improv item 2 |  |  | 0.388 (0.108) | -0.200 (0.246) |
| Improv item 4 |  |  | 0.480 (0.124) | 0.194 (0.245) |
| Improv item 5 |  |  | 0.233 (0.108) | 0.078 (0.151) |
| Improv item 6 |  |  |  | -0.001 (0.190) |
| Improv item 7 |  |  | 0.322 (0.111) | -0.066 (0.263) |
| Improv item 8 |  |  |  | -0.268 (0.158) |
| Improv item 9 |  |  |  | -0.133 (0.191) |
| Improv item 10 |  |  | 0.244 (0.110) | -0.091 (0.170) |
| Improv item 11 |  |  | 0.167 (0.104) | -0.029 (0.175) |
| Improv item 13 |  |  | 0.268 (0.111) | -0.178 (0.213) |
| Improv item 14 |  |  | 0.262 (0.122) | -0.086 (0.258) |
| Improv item 15 |  |  |  | 0.010 (0.121) |
| Improv item 16 |  |  |  | -0.294 (0.274) |
| Age * improv 8 |  |  |  | -0.130 (0.107) |
| Age * improv 14 |  |  |  | -0.128 (0.125) |
| Gender * improv 7 |  |  |  | 0.196 (0.213) |
| Improv 1 * improv 2 |  |  |  | -0.015 (0.248) |
| Improv 1 * improv 4 |  |  |  | -0.095 (0.241) |
| Improv 1 * improv 7 |  |  |  | 0.226 (0.246) |
| Improv 1 * improv 14 |  |  |  | 0.350 (0.294) |
| Improv 2 * improv 7 |  |  |  | 0.022 (0.229) |
| Improv 2 * improv 8 |  |  |  | 0.395 (0.216) |
| Improv 2 * improv 10 |  |  |  | 0.435 (0.218) |
| Improv 2 * improv 11 |  |  |  | 0.201 (0.210) |
| Improv 2 * improv 13 |  |  |  | 0.147 (0.228) |
| Improv 4 * improv 6 |  |  |  | 0.396 (0.217) |
| Improv 4 * improv 7 |  |  |  | 0.002 (0.220) |
| Improv 4 * improv 9 |  |  |  | 0.477 (0.215) |
| Improv 6 * improv 9 |  |  |  | -0.788 (0.220) |
| Improv 7 * improv 9 |  |  |  | 0.451 (0.213) |
| Improv 10 * improv 13 |  |  |  | 0.289 (0.223) |
| Improv 11 * improv 5 |  |  |  | 0.251 (0.210) |
| Improv 13 * improv 6 |  |  |  | 0.445 (0.221) |
| Improv 15 * improv 16 |  |  |  | 0.631 (0.309) |
| Observations | 1,961 | 1,961 | 1,961 | 1,961 |
| Log Likelihood | -1,315.276 | -1,249.787 | -1,195.004 | -1,164.499 |
| AIC | 2,644.551 | 2,515.574 | 2,424.008 | 2,416.998 |
| BIC | 2,683.620 | 2,560.224 | 2,518.889 | 2,662.571 |

**Supplemental table 6. Model performance in the test dataset for secondary analyses investigating interactions with treatment group**

| **Model** | **Main effect only or interactions with treatment group?** | **AUC** | | | |
| --- | --- | --- | --- | --- | --- |
|  |  | **Week 6** | | **Week 12** | |
|  |  | **Response** | **Remission** | **Response** | **Remission** |
| **Baseline** | **Main effect** | 0.61 | 0.66 | 0.63 | 0.62 |
|  | **Interactions** | 0.60 | 0.66 | 0.60 | 0.61 |
| **Total improvement** | **Main effect** | 0.74 | 0.75 | 0.69 | 0.69 |
|  | **Interactions** | 0.74 | 0.75 | 0.68 | 0.68 |
| **Item improvement** | **Main effect** | 0.78 | 0.79 | 0.72 | 0.72 |
|  | **Interactions** | 0.77 | 0.78 | 0.70 | 0.70 |
| **Item interactions** | **Main effect** | 0.78 | 0.79 | 0.72 | 0.73 |
|  | **Interactions** | 0.77 | 0.78 | 0.69 | 0.72 |

**Supplemental table 7. Model performance in the test dataset for post-hoc analyses with improvement as a continuous variable.**

| **Model** | **AUC** | | | |
| --- | --- | --- | --- | --- |
|  | **Week 6** | | **Week 12** | |
|  | **Response** | **Remission** | **Response** | **Remission** |
| **Baseline** | 0.60 | 0.63 | 0.62 | 0.63 |
| **Total improvement** | 0.79 | 0.79 | 0.71 | 0.75 |
| **Item improvement** | * | 0.79 | 0.71 | 0.74 |
| **Item interactions** | * | 0.79 | 0.71 | 0.74 |

* These models contained the same variables as the total improvement model for response at week 6.

**Supplemental table 8. Characteristics of antidepressant-treated participants who were excluded from the main analyses due to not having a week 6 or week 12 visit (“dropouts”) compared to included participants. Characteristics of all dropouts and of only those dropouts with a week 2 visit are examined.**

|  | **Week 6** | | | **Week 12** | | |
| --- | --- | --- | --- | --- | --- | --- |
|  | **Included** | **Dropouts** | | **Included** | **Dropouts** | |
|  |  | **All** | **Week 2 visit** |  | **All** | **Week 2 visit** |
| **Sample size** | 6058 | 1511 | 1018 | 2451 | 1079 | 865 |
| **Baseline HAM-D score (SD)** | 21.5 (4.5) | 22.1 (4.5) | 22.1 (4.5) | 22.5 (4.1) | 22.6 (3.9) | 22.6 (3.9) |
| **Age (SD)** | 43.8 (13.9) | 41.9 (14.6) | 42.2 (14.6) | 46.8 (15.0) | 43.5 (15.8) | 43.5 (15.8) |
| **% female** | 64 | 62 | 59 | 64 | 64 | 62 |
| **% early improvers** | 62.7 | - | 41.1 | 64.7 | - | 48.3 |

**Supplemental table 9. Model performance in the test dataset with missing week 6 or week 12 outcomes imputed.**

| **Time** | **Outcome** | **Model** | **AUC** | **Accu-racy** | **Sensi-tivity** | **Speci-ficity** | **PPV** | **NPV** |
| --- | --- | --- | --- | --- | --- | --- | --- | --- |
| Week 6 | Response | Baseline | 0.60 | 0.57 | 0.54 | 0.60 | 0.58 | 0.56 |
|  |  | Total improvement | 0.72 | 0.68 | 0.76 | 0.60 | 0.66 | 0.71 |
|  |  | Item improvement | 0.76 | 0.70 | 0.70 | 0.69 | 0.70 | 0.69 |
|  |  | Item interactions | 0.76 | 0.69 | 0.68 | 0.71 | 0.70 | 0.68 |
|  | Remission | Baseline | 0.62 | 0.71 | 0.10 | 0.97 | 0.63 | 0.71 |
|  |  | Total improvement | 0.73 | 0.72 | 0.29 | 0.91 | 0.59 | 0.75 |
|  |  | Item improvement | 0.78 | 0.74 | 0.41 | 0.89 | 0.61 | 0.78 |
|  |  | Item interactions | 0.77 | 0.74 | 0.41 | 0.88 | 0.61 | 0.78 |
| Week 12 | Response | Baseline | 0.61 | 0.66 | 0.96 | 0.09 | 0.67 | 0.54 |
|  |  | Total improvement | 0.70 | 0.69 | 0.90 | 0.29 | 0.71 | 0.61 |
|  |  | Item improvement | 0.73 | 0.70 | 0.87 | 0.37 | 0.72 | 0.60 |
|  |  | Item interactions | 0.72 | 0.69 | 0.86 | 0.38 | 0.72 | 0.58 |
|  | Remission | Baseline | 0.62 | 0.59 | 0.50 | 0.68 | 0.58 | 0.60 |
|  |  | Total improvement | 0.71 | 0.65 | 0.61 | 0.68 | 0.63 | 0.66 |
|  |  | Item improvement | 0.75 | 0.68 | 0.65 | 0.71 | 0.66 | 0.69 |
|  |  | Item interactions | 0.75 | 0.69 | 0.62 | 0.74 | 0.68 | 0.69 |

**Supplemental figures**

**
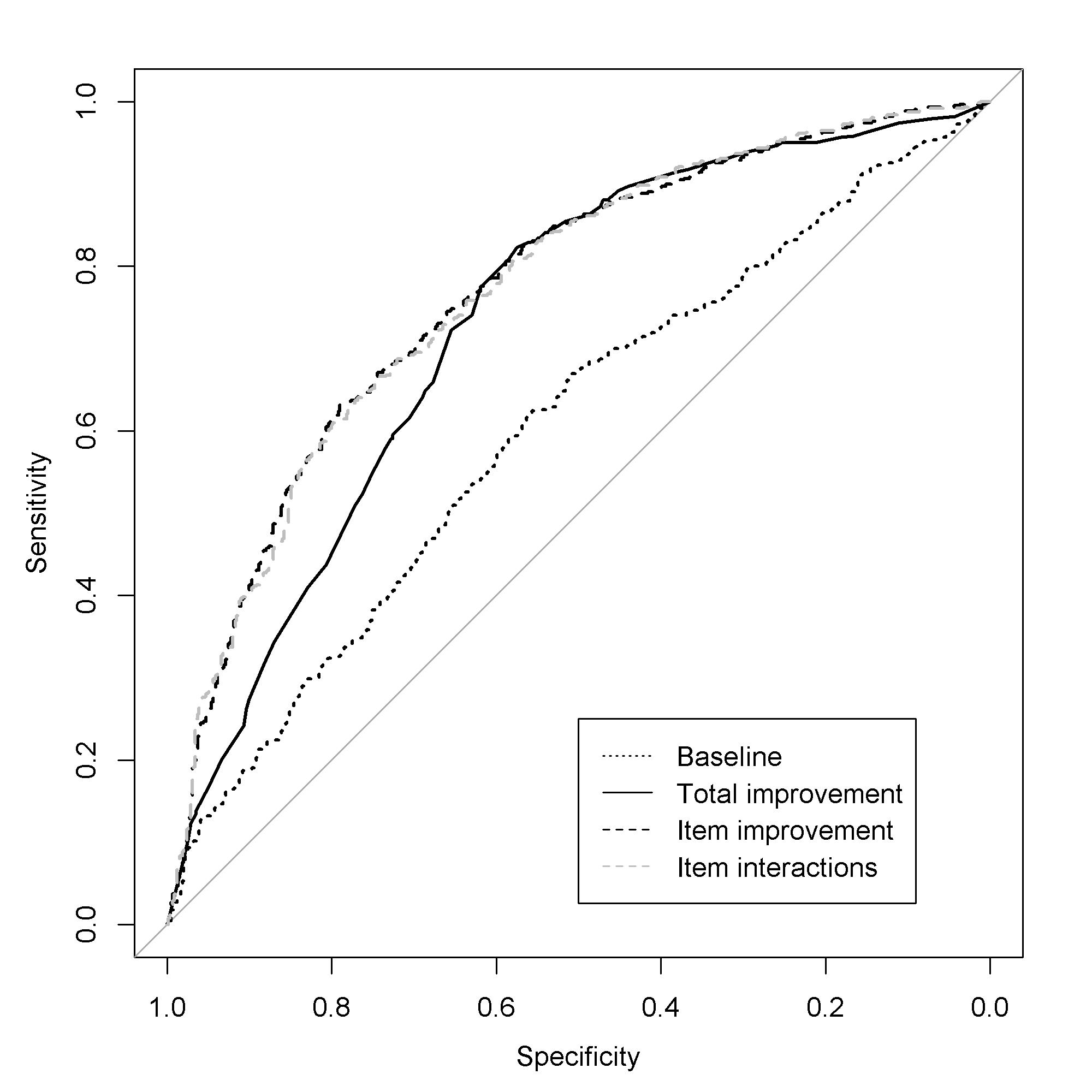
**

Supplemental figure 1: Receiver-operating characteristic curve for the baseline, total improvement, item improvement, and item interactions model for response at week 6 in the test dataset.

**
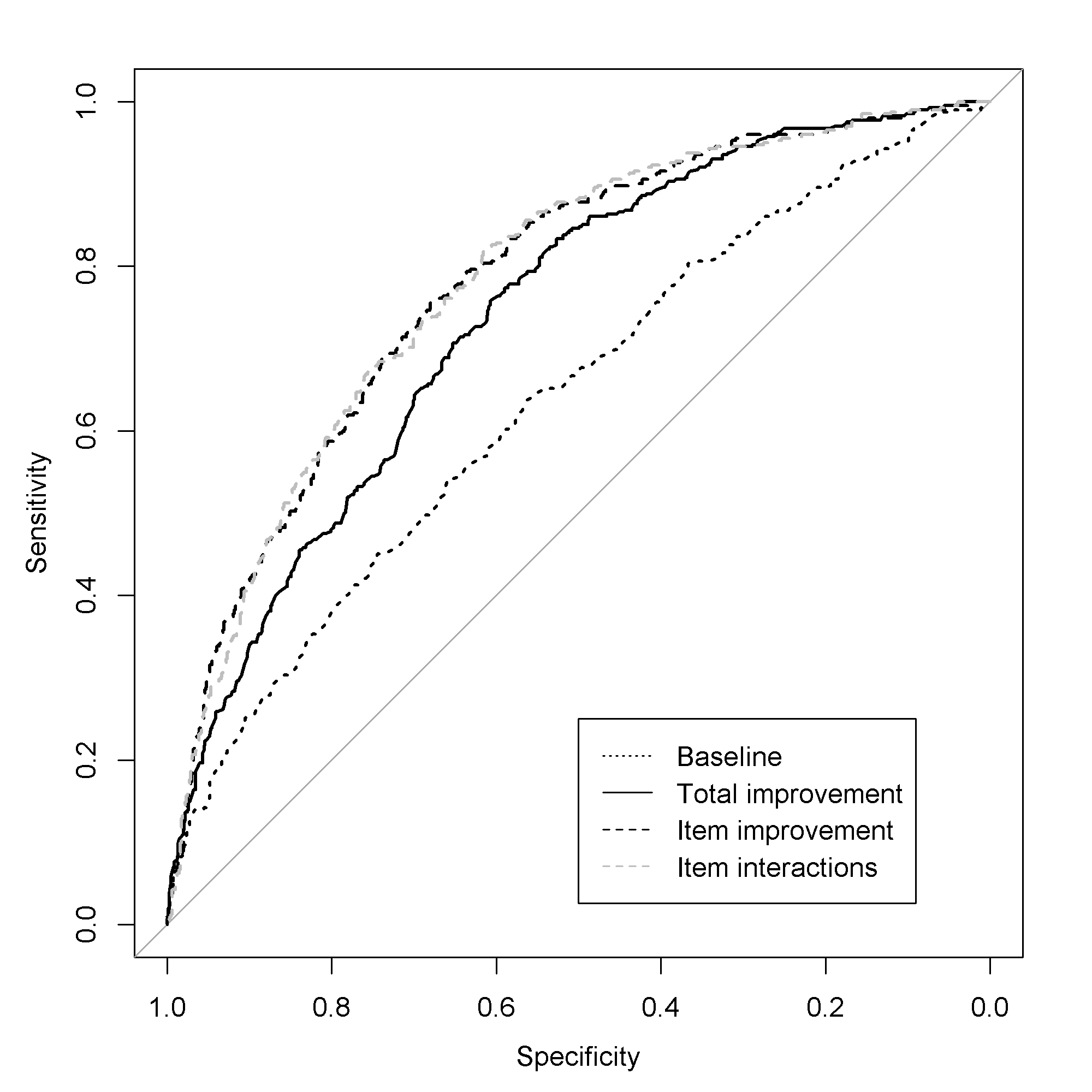
**

Supplemental figure 2: Receiver-operating characteristic curve for the baseline, total improvement, item improvement, and item interactions model for remission at week 6 in the test dataset.

**
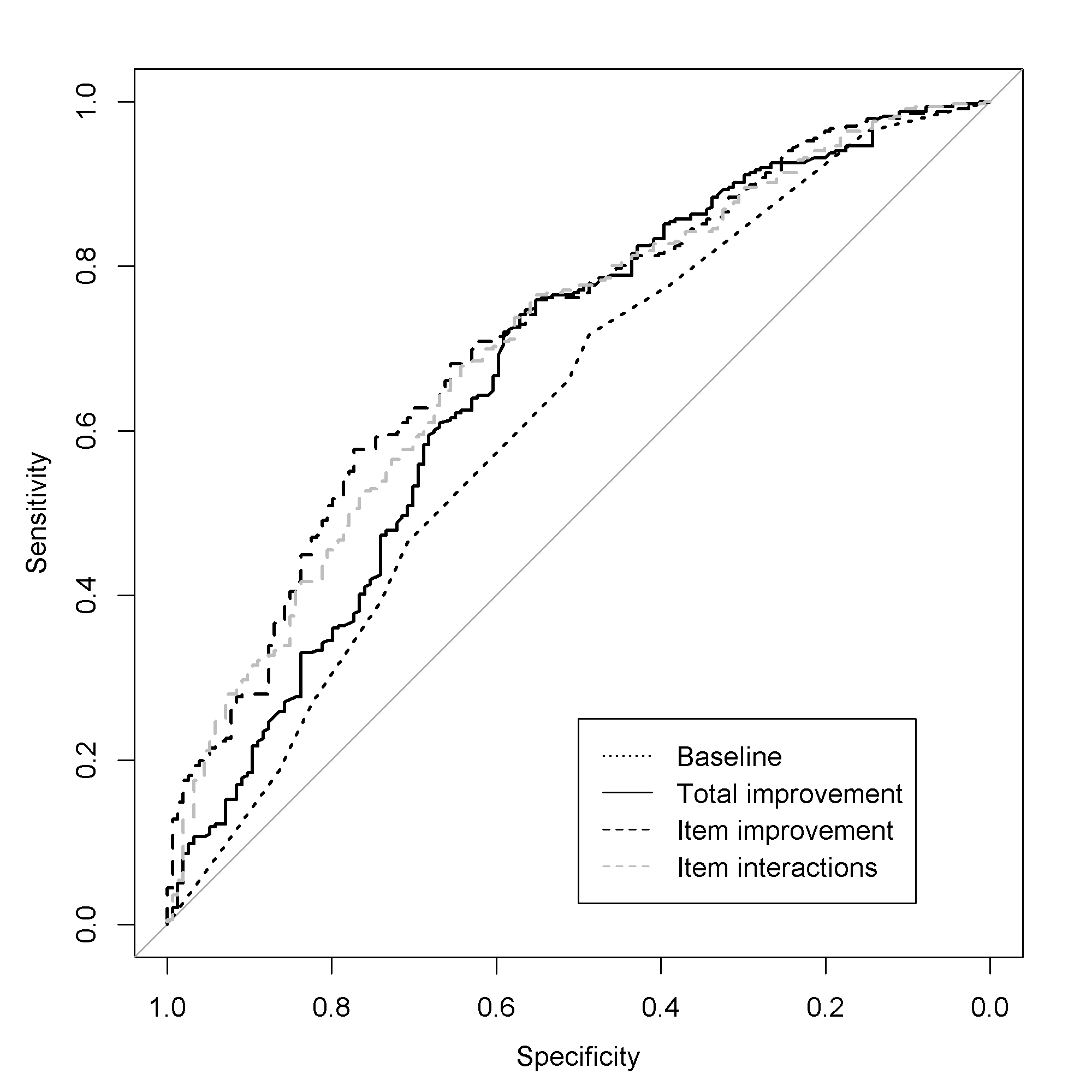
**

Supplemental figure 3: Receiver-operating characteristic curve for the baseline, total improvement, item improvement, and item interactions model for response at week 12 in the test dataset.

**
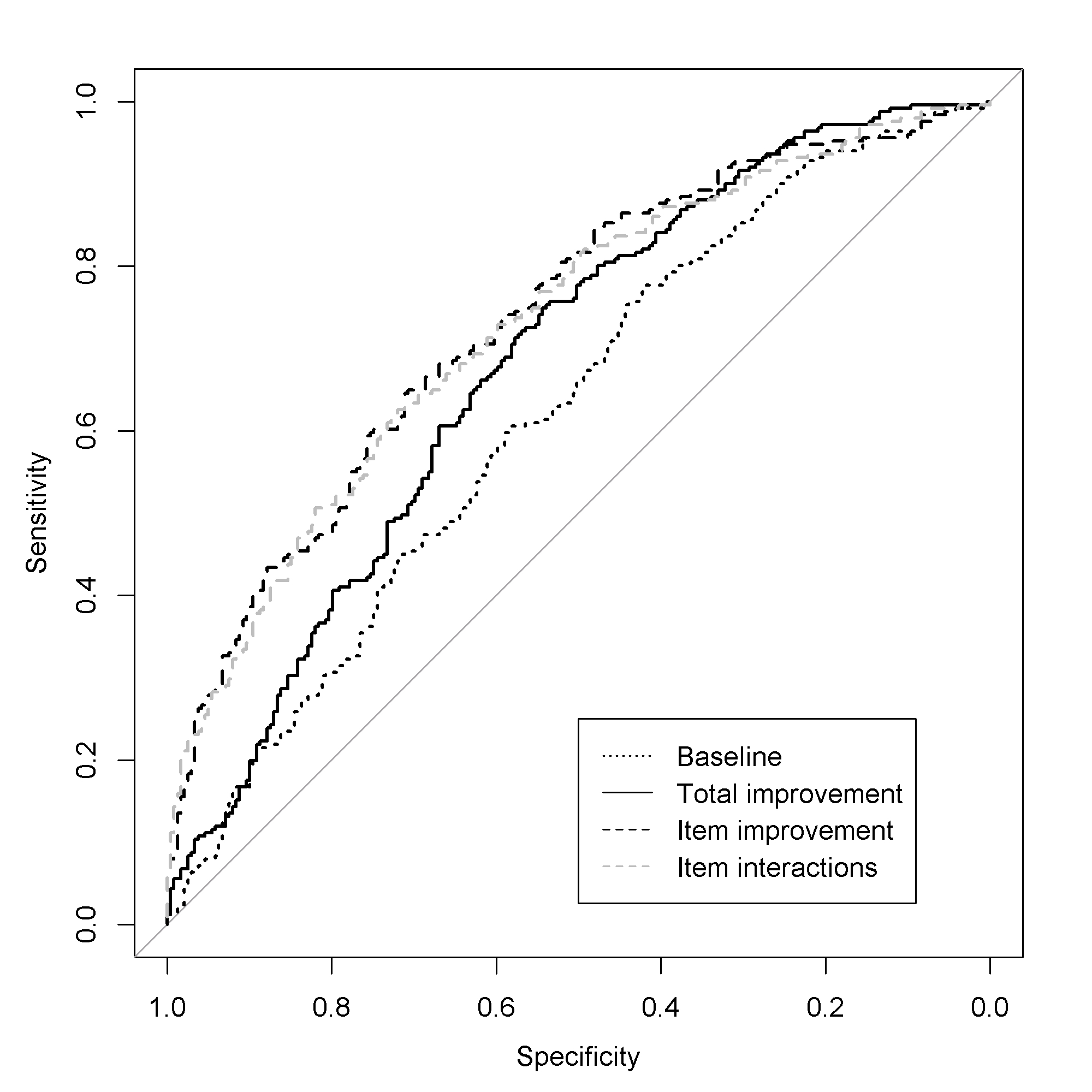
**

Supplemental figure 4: Receiver-operating characteristic curve for the baseline, total improvement, item improvement, and item interactions model for remission at week 12 in the test dataset.

**
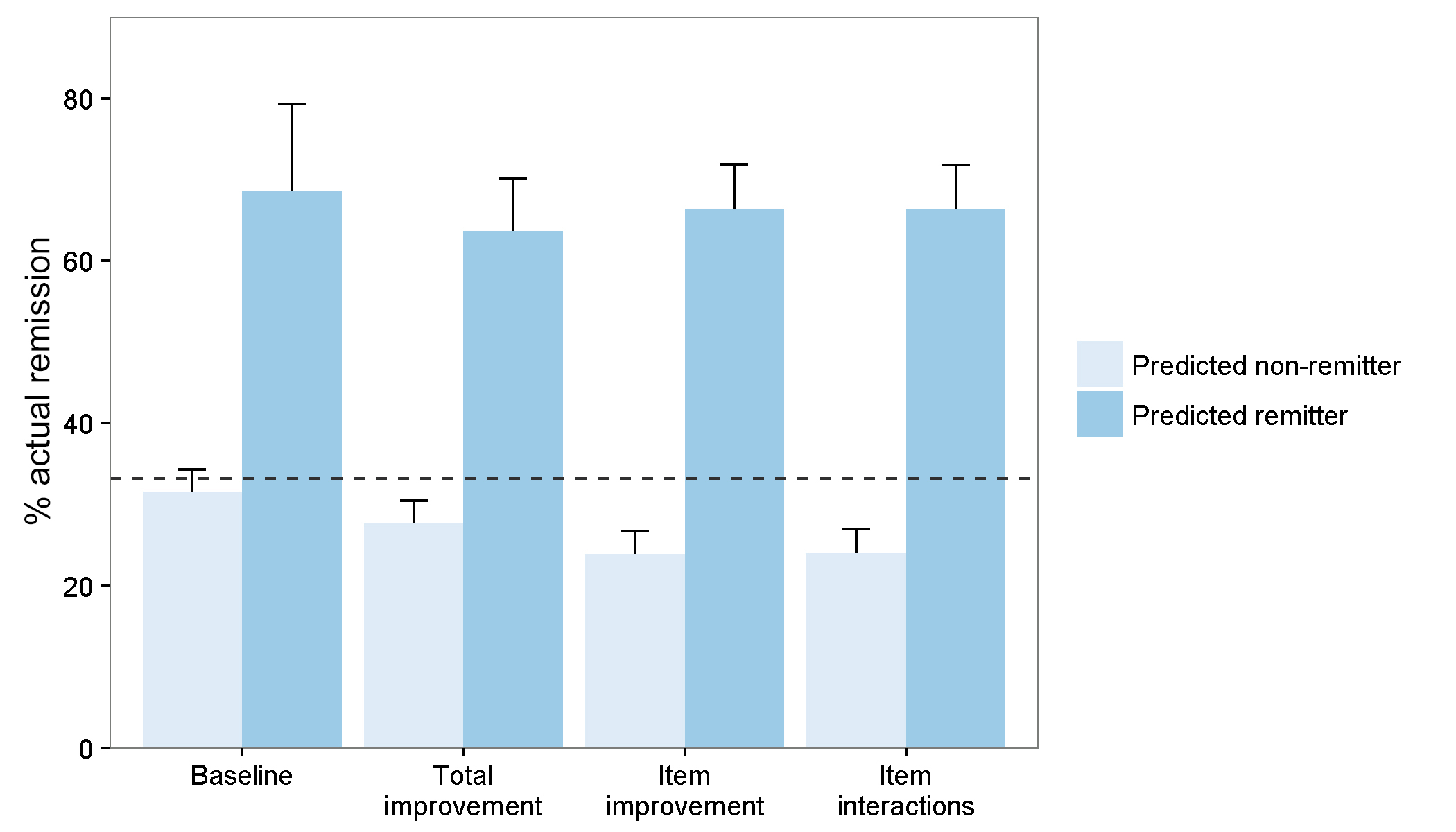
**

Supplemental figure 5: Actual probability of remission at week 6 according to participants’ predicted outcome (non-remission vs. remission) in the test dataset. The dashed line indicates the baseline probability of remission. The models predicted non-remission for 96% (baseline), 85% (total improvement), 78% (item improvement), and 78% (item interactions) of participants.

**
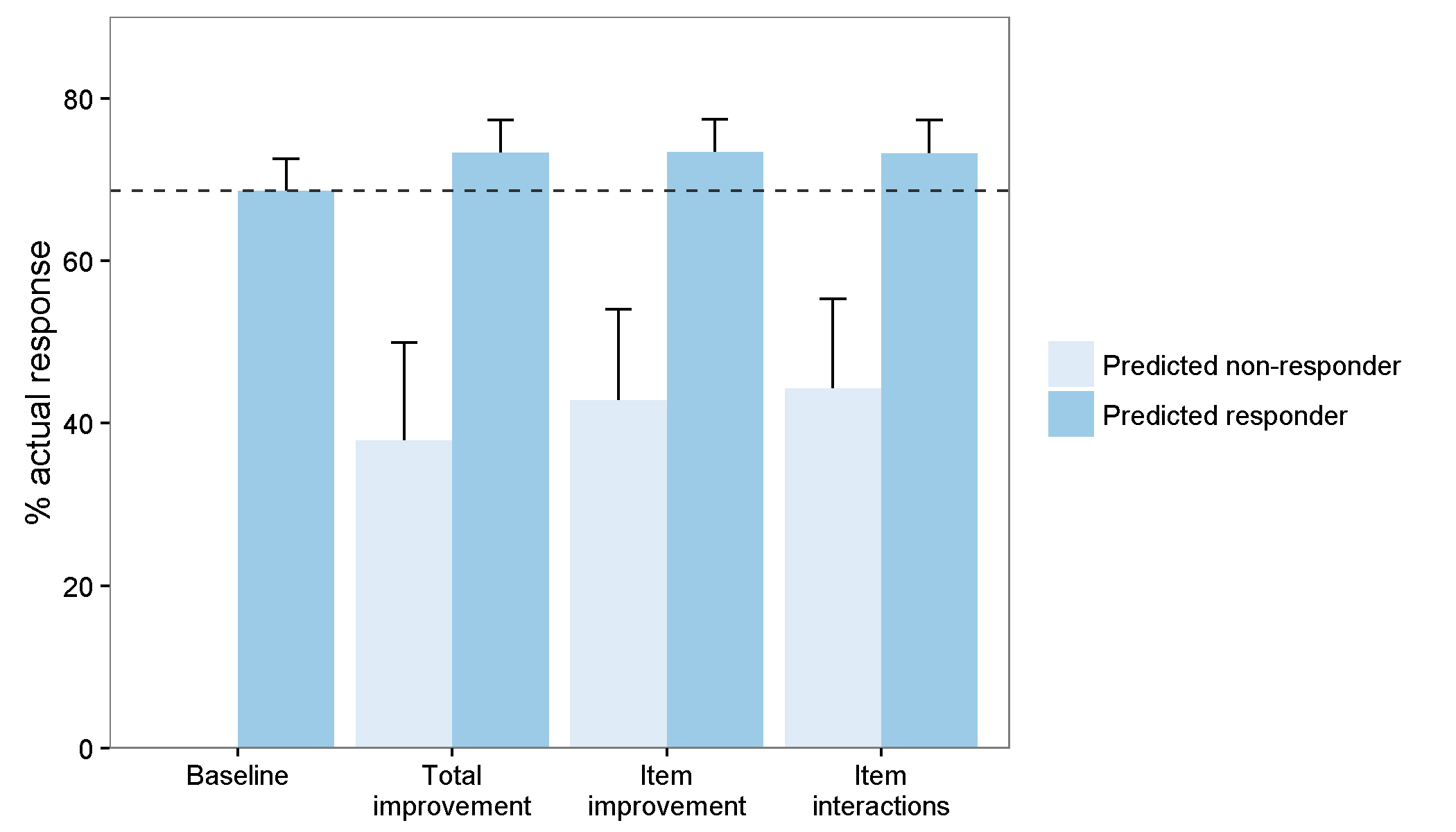
**

Supplemental figure 6: Actual probability of response at week 12 according to participants’ predicted outcome (non-response vs. response) in the test dataset. The dashed line indicates the baseline probability of response. The models predicted non-response for 0% (baseline), 13% (total improvement), 16% (item improvement), and 16% (item interactions) of participants.

**
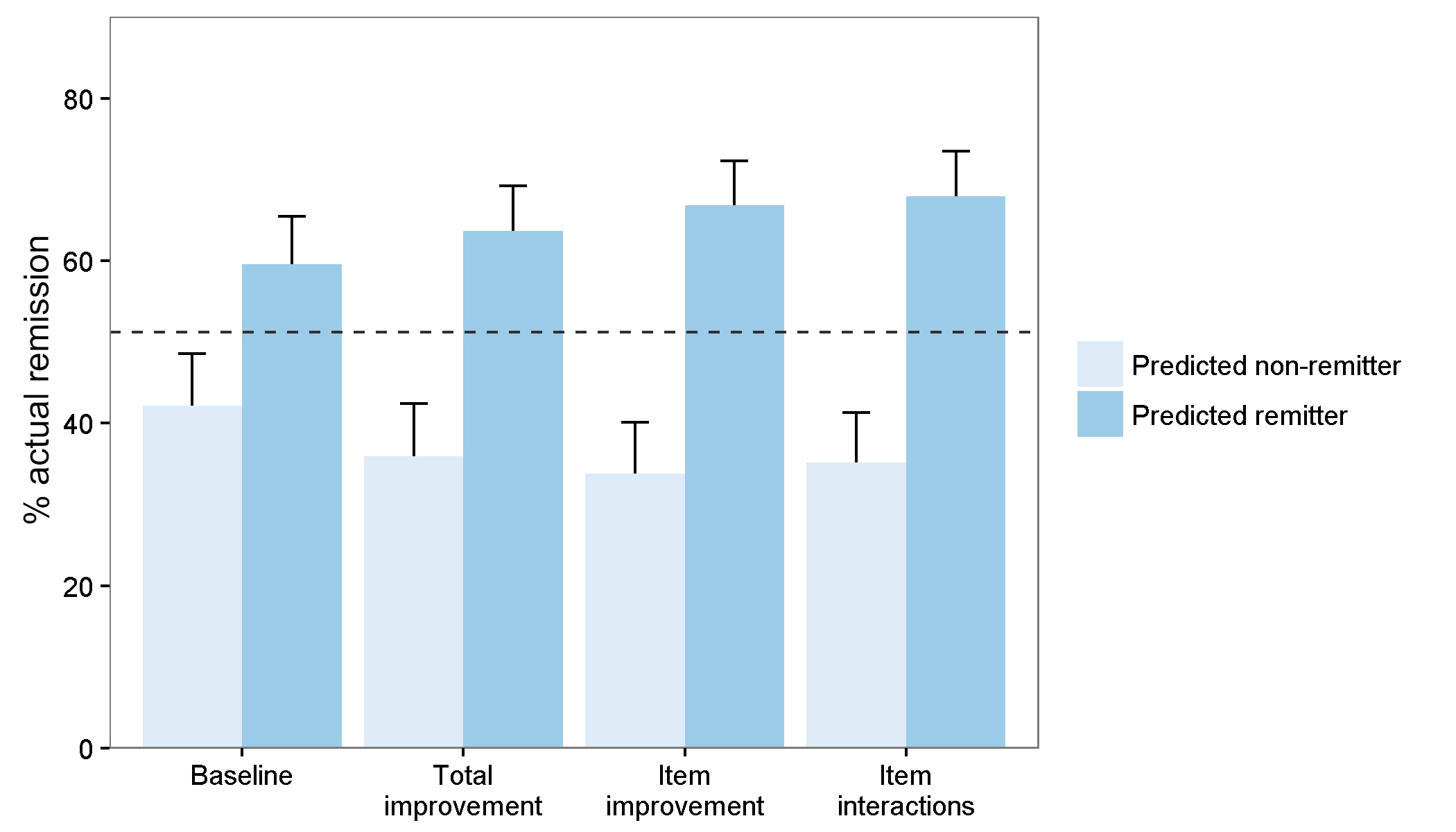
**

Supplemental figure 7: Actual probability of remission at week 12 according to participants’ predicted outcome (non-remission vs. remission) in the test dataset. The dashed line indicates the baseline probability of remission. The models predicted non-remission for 48% (baseline), 45% (total improvement), 47% (item improvement), and 51% (item interactions) of participants.

**
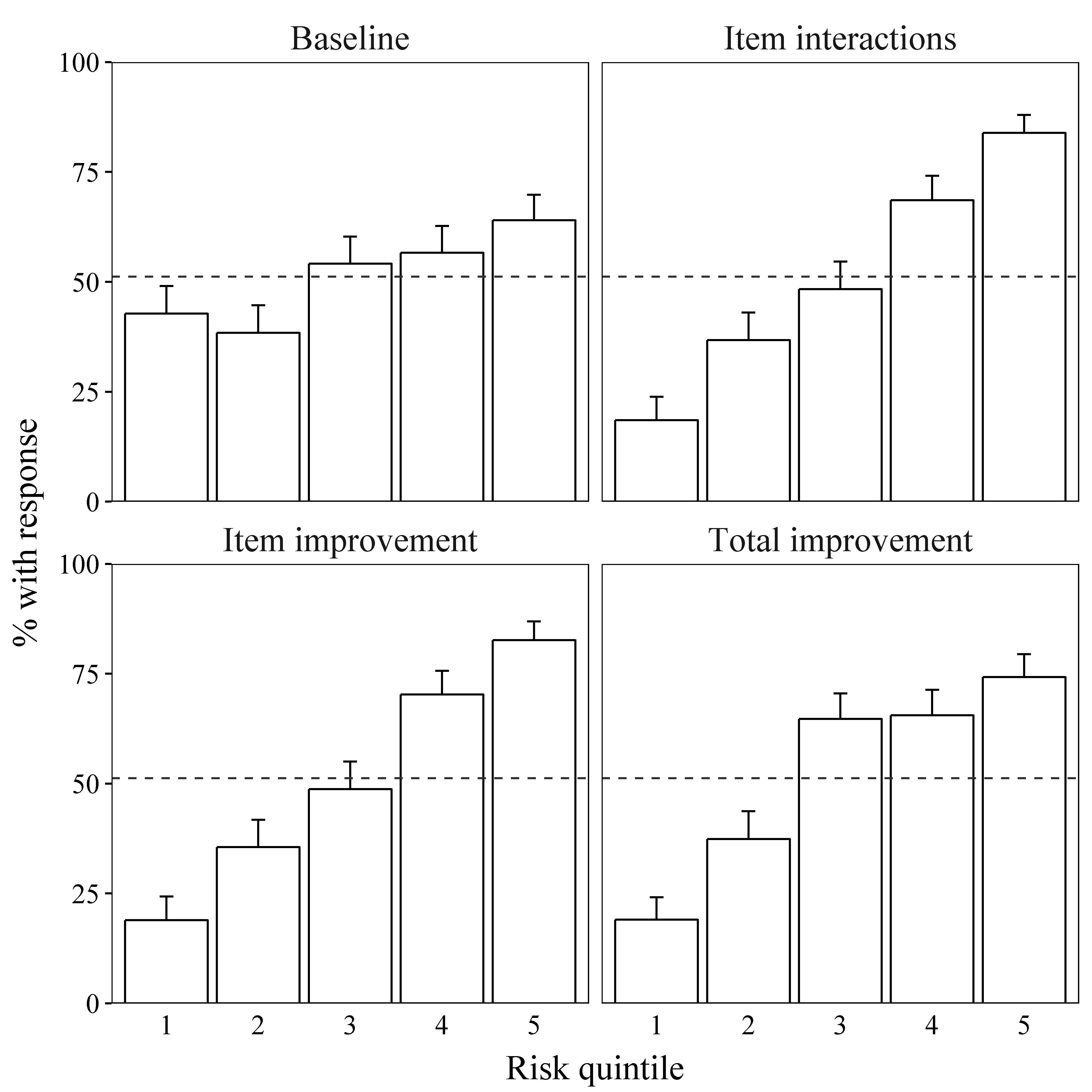
**

Supplemental figure 8: Actual probability of response at week 6 according to risk quantiles and model in the test dataset. For each model, participants’ predicted probability of response was used to divide participants into quintiles of “risk”, with the lowest quintile having the lowest predicted probability of response.

**
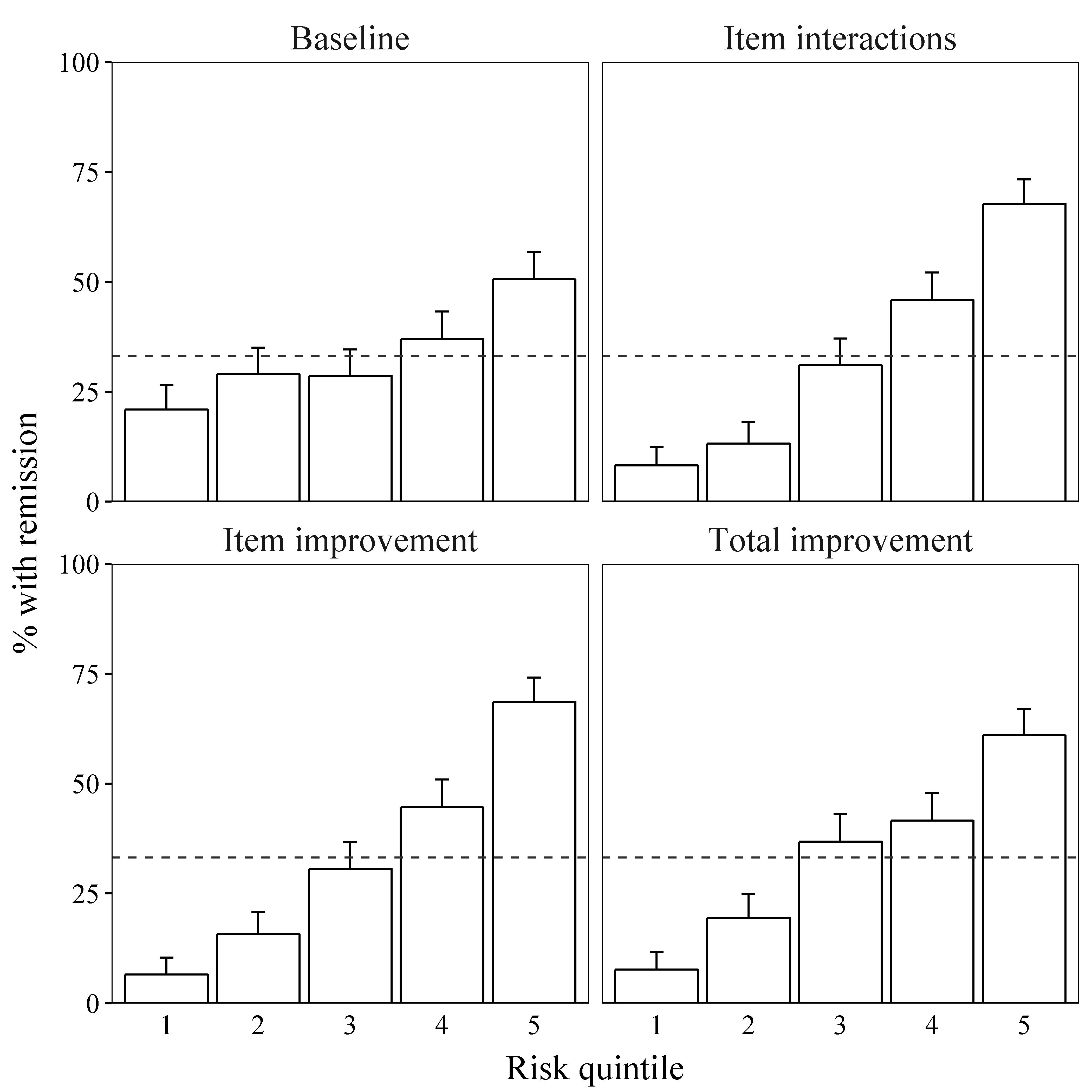
**

Supplemental figure 9: Actual probability of remission at week 6 according to risk quantiles and model in the test dataset. For each model, participants’ predicted probability of remission was used to divide participants into quintiles of “risk”, with the lowest quintile having the lowest predicted probability of remission.

**
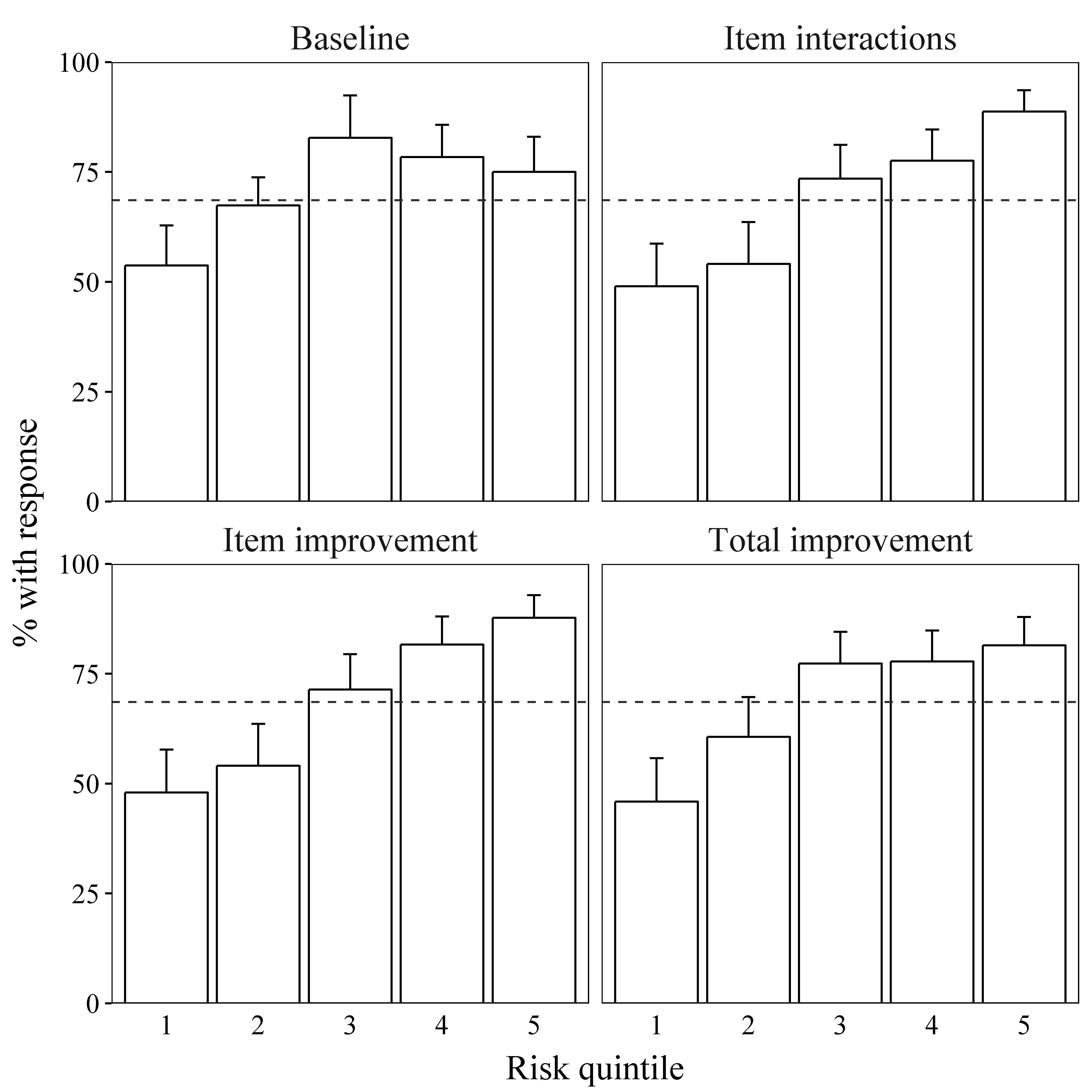
**

Supplemental figure 10: Actual probability of response at week 12 according to risk quantiles and model in the test dataset. For each model, participants’ predicted probability of response was used to divide participants into quintiles of “risk”, with the lowest quintile having the lowest predicted probability of response.

**
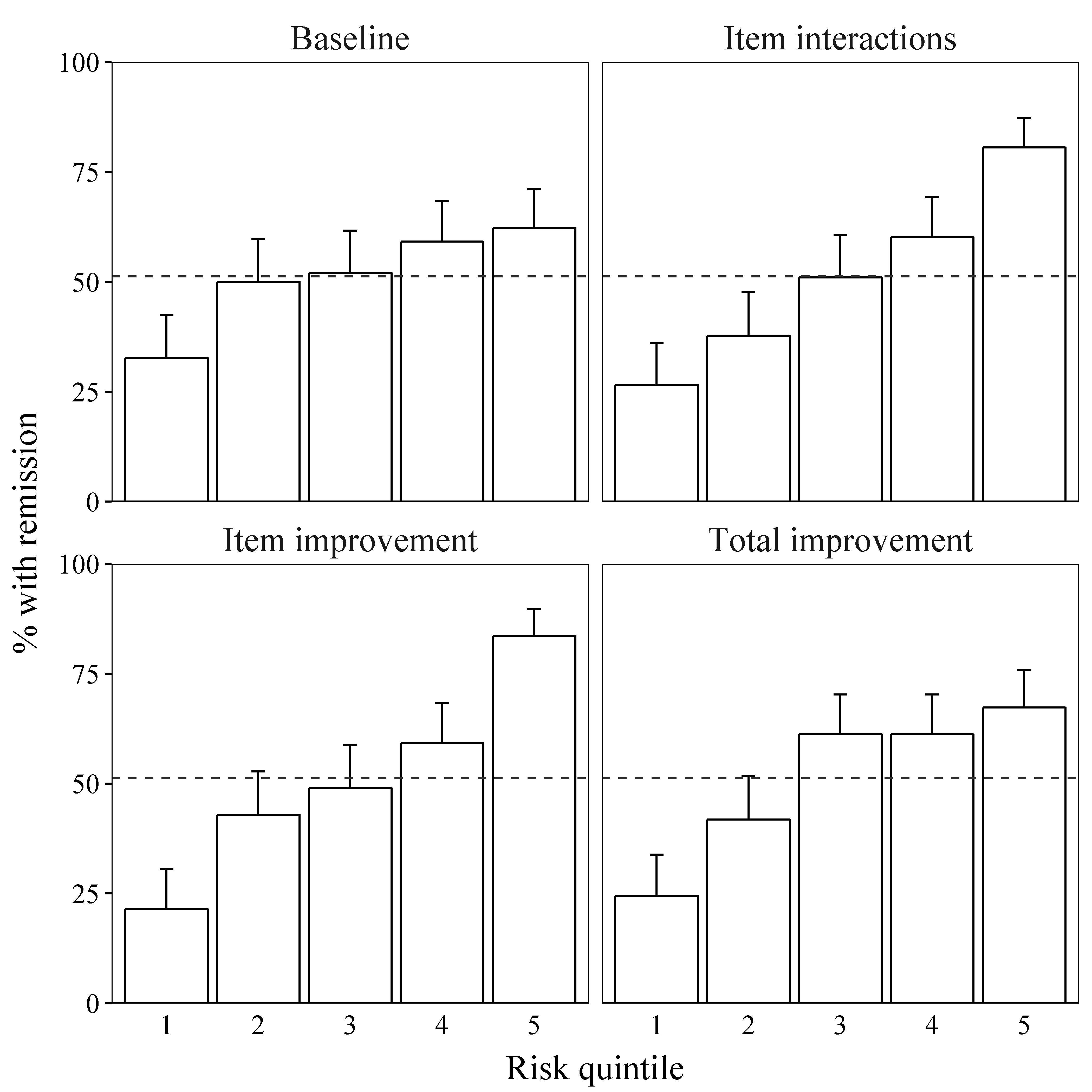
**

Supplemental figure 11: Actual probability of remission at week 12 according to risk quantiles and model in the test dataset. For each model, participants’ predicted probability of remission was used to divide participants into quintiles of “risk”, with the lowest quintile having the lowest predicted probability of remission.
